# Plaque-targeted, proteolysis-resistant, activatable and MRI-visible nano-GLP-1 receptor agonist targets smooth muscle cell differentiation in atherosclerosis

**DOI:** 10.7150/thno.66456

**Published:** 2022-03-06

**Authors:** Andrei Maiseyeu, Lin Di, Anastasia Ravodina, Alma Barajas-Espinosa, Atsushi Sakamoto, Alice Chaplin, Jixin Zhong, Huiyun Gao, Matthew Mignery, Navneet Narula, Aloke V. Finn, Sanjay Rajagopalan

**Affiliations:** 1 Case Western Reserve University, Cleveland, OH; 2 Dorothy M. Davis Heart and Lung Research Institute, The Ohio State University, Columbus, OH; 3 University of Maryland, Baltimore, MD; 4 CVPath Institute, Inc., Gaithersburg, MD; 5 NYU Langone Medical Center, New York, NY

**Keywords:** GLP-1, atherosclerosis, nanomedicine, MRI, smooth muscle cells

## Abstract

**Background:** Glucagon-like peptide-1 receptor (GLP-1R) agonists are powerful glycemia-lowering agents, which have systematically been shown to lower cardiovascular events and mortality. These beneficial effects were difficult to pinpoint within atherosclerotic plaque due to lack of particular specificity of such agonists to the vascular cells and an inadequate understanding of the GLP-1R expression in atherosclerosis. Here, we hypothesized that the direct engagement of the GLP-1R in atherosclerosis by targeted agonists will alleviate vascular inflammation and plaque burden, even at a very low dose.

**Methods:** The expression of GLP-1 receptor (GLP-1R, *Glp1r* mRNA) in human lesions with pathologic intimal thickening, Apoe^-/-^ mouse atheroma and cultured immune/non-immune cells was investigated using genetic lineage tracing, Southern blotting and validated antisera against human GLP-1R. Protease-resistant and “activatable” nanoparticles (NPs) carrying GLP-1R agonist liraglutide (GlpNP) were engineered and synthesized. Inclusion of gadolinium chelates into GlpNP allowed for imaging by MRI. Atherosclerotic Apoe^-/-^ mice were treated intravenously with a single dose (30 µg/kg of liraglutide) or chronically (1 µg/kg, 6 weeks, 2x/week) with GlpNP, liraglutide or control NPs, followed by assessment of metabolic parameters, atheroma burden, inflammation and vascular function.

**Results:** Humal plaque specimens expressed high levels of GLP-1R within the locus of de-differentiated smooth muscle cells that also expressed myeloid marker CD68. However, innate immune cells under a variety of conditions expressed very low levels of *Glp1r*, as seen in lineage tracing and Southern blotting experiments examining full-length open reading frame mRNA transcripts. Importantly, de-differentiated vascular smooth muscle cells demonstrated significant *Glp1r* expression levels, suggesting that these could represent the cells with predominant *Glp1r-*positivity in atherosclerosis. GlpNP resisted proteolysis and demonstrated biological activity including *in vivo* glycemia lowering at 30 µg/kg and *in vitro* cholesterol efflux. Activatable properties of GlpNP were confirmed* in vitro* by imaging cytometry and *in vivo* using whole organ imaging. GlpNP targeted CD11b^+^/CD11c^+^ cells in circulation and smooth muscle cells in aortic plaque in Apoe^-/-^ mice when assessed by MRI and fluorescence imaging. At a very low dose of 1 µg/kg, previously known to have little effect on glycemia and weight loss, GlpNP delivered i.v. for six weeks reduced triglyceride-rich lipoproteins in plasma, plaque burden and plaque cholesterol without significant effects on weight, glycemia and plasma cholesterol levels.

**Conclusions**: GlpNP improves atherosclerosis at weight-neutral doses as low as 1 µg/kg with the effects independent from the pancreas or the central nervous system. Our study underlines the importance of direct actions of GLP-1 analogs on atherosclerosis, involving cholesterol efflux and inflammation. Our findings are the first to suggest the therapeutic modulation of vascular targets by GlpNP, especially in the context of smooth muscle cell inflammation.

## Introduction

Glucagon like peptide-1 receptor (GLP-1R) agonists have been shown to have an important role in reducing major adverse cardiovascular events 1. However, the mechanisms of action have been difficult to unravel given the pleiotropic impact of these agents on the cardiovascular system, including weight loss, reduction in atherogenic lipoproteins and inflammation, as well as blood pressure lowering 2. *In vitro* studies and experimental animal models with GLP-1R agonists have demonstrated consistent reduction in atherosclerosis which have been theorized, at least in part, to be mediated through GLP-1R and cAMP signaling pathways 3. This benefit on atherosclerosis has resulted in the implicit assumption that the GLP-1R is expressed widely in the cardiovascular system. However, a number of studies primarily in non-atherosclerotic models and in heart transplant recipients have demonstrated a rather restricted spatial expression of the receptor in the vasculature and myocardium, where a low levels of *Glp1r* mRNA transcripts were found in specific cells including smooth muscle cells and atrial cell populations 4,5. Importantly, examining full-length *Glp1r* mRNA encoding for GLP-1R open reading frame remains the only reliable method for investigation of the *Glp1r* expression in rodent tissues and cells, as many commercially available antibodies proved to be non-specific 5,6.

Here, we set out to investigate whether plaque-specific delivery of ultra-small, targeted doses of GLP-1R may allow decoupling of systemic effects of GLP-1R activation from those that may be spatially restricted to the vascular compartment. In a proof-of-concept study we show that the delivery of microdoses of *Glp1r* agonist by means of “activatable” nanoparticles, via an engineered drug delivery vehicle enables spatially restricted and site-specific activation in atherosclerosis. Additionally, we investigated the cell-specific expression and localization of *Glp1r* in the atherosclerotic plaque, plaque-derived cells and cultured primary cells together with coordinate experiments in human atherosclerosis.

## Methods

Detailed methods are provided in the Supplementary material online. Specific identifiers, such as catalog numbers, of reagents, supplies and materials used in this study are shown in Supplementary Table S1.

### Animals

Mice homozygous for the Apoe^tm1Unc^ mutation were purchased from The Jackson Laboratory (Bar Harbor, ME). Glp1^-/-^ and Glp1r-Cre/ROSA26R-YFP were obtained from Dr. Jixin Zhong's laboratory and generated as previously described 7. Mice were housed in AAALAC-accredited facilities at Case Western Reserve University, University of Maryland, and Ohio State University. The Institutional Animal Care and Use Committee (IACUC) approved the experimental procedures described herein. Mice were purchased at 10 weeks old and acclimated in the facility for 2 weeks, following which a high-fat 1.25 % cholesterol diet (Research Diets, New Brunswick, NJ) was given *ad libitum* (western diet, WD*)*. In chronic GlpNP treatment studies, male mice were fed for 24 weeks followed by randomization and treatment. Nine mice were in liraglutide, eight mice in GlpNP, and seven in the control group. In *Glp1r* expression determination experiments, 10 mice were fed WD for 15 weeks and 11 mice were fed WD for 30 weeks. Eight littermate controls were fed a chow diet for 30 weeks. For atherosclerosis studies, liraglutide or nanoparticles were administered via tail-vein or penile-vein injections at 30 µg/kg (based on liraglutide) in a single dose experiment and 1 µg/kg /two times a week for six weeks in chronic experiments. For baseline sample collection, mice were fasted for 12 h and 20-50 µL of blood was drawn from the tail nick. Blood was analyzed as described below. In terminal chronic experiments, mice were euthanized by CO_2_ asphyxiation followed by cervical dislocation. The methods for euthanasia used are consistent with the recommendations of the Panel on Euthanasia of the American Veterinary Medical Association (AVMA). Accordingly, animals were euthanized by CO_2_ asphyxiation at gas flow rates recommended by AVMA as follows: displacing no more than 30% of the chamber volume per minute in euthanasia apparatus. This method allows for slow onset CO_2_ narcosis followed by death and is more humane than higher flow rates. Secondary euthanasia method (cervical dislocation) was always used to ensure non-revival. Blood was drawn by cardiac puncture and aortas were collected either in optimal cutting temperature compound (for laser capture) or in buffered 4% formaldehyde (for immunohistochemistry). Liver and other tissues were snap-frozen in liquid nitrogen and kept at -80 ^o^C until RNA and lipid isolation for further analysis.

### Primary mouse aortic smooth muscle cells (MASMCs)

MASMCs were obtained from 4-6 week old Apoe^-/-^ female mouse aortas as previously described 8. Briefly, aortas were cleaned and digested in a cocktail consisting of 1 mg/mL collagenase II, 1 mg/mL soybean trypsin inhibitor, and 0.7 U/mL elastase in Hank's balanced salt solution with calcium and magnesium (HBSS) for 10 min at 37°C and 5% CO_2_. The adventitia was then removed under a dissecting microscope, and endothelial cells were scraped off using a pipet tip to expose the luminal surface. The 4-5 aortas were pooled and minced into <1 mm pieces that were further digested for 1 h at 37°C and 5% CO_2_ to release MASMCs. The cells were then cultured in a 48-well plate at a density of cells from 2 aortas/well in DMEM-F-12 containing 20% FBS, 1% penicillin-streptomycin, and 1 mM GlutaMAX. When the cells were 80-90% confluent, they were passaged into a 24-well Corning Primaria plate at a 1:4 ratio and treated as indicated below when ~70% confluent. This was followed by cell lysis in TRIzol reagent and RNA isolation. In some experiments, the MASMCs were seeded in Ibidi 8-well chamber slides followed by treatment, fixation, staining (see below) and immunofluorescence analysis. MASMCs were used between passages 2 and 3 in all experiments.

### Cell treatment

BMDMs, MASMCs and 3B11 were switched from normal culture medium to serum-free DMEM containing 0.1% BSA. Primary endothelial cells were cultured in complete medium. The following treatments were applied for 48 h: 10 µg/mL cholesterol water-soluble (Chol), 2 µg/mL CP-113,818 (ACATi), combination of both Chol and ACATi, 100 nM liraglutide, combination of three: Chol, ACATi and liraglutide. Separately, 100 µg/mL of 2 µm polystyrene latex beads (Beads) were also used. In separate experiments, modified lipoproteins were added to BMDMs at a concentration of 0.5 µg/mL for 48 h. UV-induced apoptotic bodies (UV-AB) were generated by exposure of viable Jurkat cells to UV light (254 nm) for 5 min followed by culture at 37^o^C until 30-50% viable as per trypan blue exclusion test. These UV-AB were then added to BMDMs at 10^4^ dead cells per 10^6^ of viable BMDMs and incubated for 48 h. For TGFβ treatment of bone-marrow monocytes, the bone marrow cells were isolated in the same manner as for BMDM differentiation and cultured in the presence of 40 ng/mL of macrophage colony-stimulating factor (MCSF) for 2 days. This was followed by the withdrawal of MCSF and the addition of mouse recombinant TGFβ at 1 ng/mL. These cells were cultured for an additional 4 days with a new medium containing fresh TGFβ replenished daily. For TGFβ treatment of primary mouse carotid artery endothelial cells, the cells were treated in complete medium supplemented with 50 ng/mL of mouse recombinant TGFβ and cultured for seven days with a new medium containing fresh TGFβ replenished daily.

### Southern blotting

At least 200 ng of total RNA from the tissue or cell lysates was isolated, digested with DNAse I to remove genomic DNA and purified using Zymo Clean & Concentrator kit as per manufacturer's instructions. cDNA synthesis and PCR amplification were performed using Superscript IV and SuperFi II high fidelity polymerase respectively and as per the manufacturer's instructions (ThermoFisher Scientific). The sequences for primers and probes used in PCR and hybridization are presented in Supplementary Table S1 and were custom-synthesized according to previously described sequences 9. PCR products resulting from 35 amplification cycles (for both *Glp1r* and *Hprt*) were loaded on 1% agarose gel and resolved in borate-lithium acetate buffer pH 7 at 200 V. The products were transferred overnight at room temperature to a positively-charged nylon membrane using a standard downward transfer 10 in a 10xSSC buffer (see below). The membranes were then cross-linked (UV light, 5 min, 254 nm) using Azure C400 transilluminator, washed with water and dried at 60 ^o^C for 30 min. Rolled membranes were placed in a hybridization tubes and pre-hybridized for 1 h at 44^o^C in a hybridization oven in a hybridization buffer consisting of 5xSSC, 1% dextran sulfate, 4 M urea and 1% sodium dodecyl sulfate (SDS). 20xSSC contained 3 M NaCl and 300 mM sodium citrate, pH 7.0 and was diluted with distilled water as required. After pre-hybridization, the membranes were hybridized for 18 h at 44^o^C in the same buffer with 1 pM internal digoxigenin-labeled probe (see Supplementary Table S1). Next, the membranes were subjected to stringency washes: twice under a low stringency (2xSSC, 0.1% SDS, 5 min, room temperature) and three times under a high stringency (0.1xSSC, 1% SDS, 30 min, 44^o^C) conditions. After stringency washes, the membranes were washed and blocked using DIG Wash and Block Buffer Set (Roche/Sigma-Aldrich) according to manufacturer's recommendations. Finally, the membranes were immunoprobed with a polyclonal anti-digoxigenin alkaline phosphatase-conjugated antibody at 1:20,000 dilution and developed with CDP-star chemiluminescent substrate (both from Roche/Sigma-Aldrich), according to the manufacturer's instructions. The signal was recorded using Azure C400 imager and quantified with Azure Spot software. Agarose gels containing *Hprt*-amplified PCR products were stained with SYBR Gold nucleic acid gel stain applied at 1:10,000 in borate-lithium acetate buffer pH 7 for 30 min, were imaged using UV transilluminator of Azure C400 and quantified with Azure Spot software. The chemiluminescence signal from southern hybridization was normalized to the signal from SYBR Gold-stained gels.

### Immunofluorescence studies

MASMCs were seeded in chambered coverslips (Ibidi GmbH) as per manufacturer's instructions. After cells were attached, the cells were treated as described above and in the figure legend. The cells were washed with PBS three times and fixed in 2% buffered formalin for 10 min at 4^o^C. The cells were incubated in a blocking buffer consisting of 50% fish serum (Aqua block) and 0.2% Triton X100 in PBS for 30 min at room temperature. Next, the cells were probed overnight at 4^o^C with primary antibodies against mouse ɑSMA mAb at a 1:500 dilution, rabbit SM22 and SM-MHC pAbs both at a 1:200 dilution, rat Mac3 (Cd107b) mAb at a 1:30 dilution, and rat LGALS3 (galectin-3) mAb at a 1:50 dilution, GLP-1R mAb 7F38 at a 1:30 dilution in 50% Aqua block containing 0.1% Triton X100. After five washes with cold PBS, the cells were incubated with secondary antibodies: goat anti-rat IgG Alexa Fluor 594 for Mac3 and galectin-3 mAbs-treated coverslips, goat anti-mouse IgG Alexa Fluor Plus 488 for ɑSMA treated coverslips, goat anti-rabbit IgG Alexa Fluor Plus 594 for SM22 and SM-MHC treated coverslips and goat anti-mouse IgG2a (isotype-specific) Alexa Fluor 647. All secondary antibodies were used at a 1:250 dilution from a 2 mg/mL stock and the incubation proceeded for 2 h at a room temperature in the dark. During the last 10 min of incubation, Hoechst 33342 was added at 10 µg/mL. The coverslips were washed five times with PBS and imaged immediately with Olympus FV1200 confocal microscope or Keyence BZX-700 fluorescence microscope.

For immunofluorescence studies in mouse brachiocephalic artery (BCA) and aortic root, 10 µm pre-dried frozen sections were fixed by immersion in acetone for 10 min at -20^o^C, drying and rehydrating. Blocking and staining with primary and fluorochrome-conjugated secondary antibodies was accomplished as above for chambered coverslips, with the exception that Triton X100 was omitted from incubation buffers. Antibodies were LGALS3 used at 1:50 dilution and ɑSMA used at 1:200 dilution or goat anti-ɑSMA used at 1:500 dilution. GLP-1R mAb 7F38 was used at a 1:20 dilution. Secondary antibodies were used as described above for cultured MASMCs staining. Vectashield mounting medium with DAPI was used to stain nuclei and mount the slides.

### Nanoparticle and GlpNP synthesis and analysis

GlpNP and control nanoparticles (with and without PtdSer or liraglutide) were prepared by vesicle fusion and screened using different concentrations of phosphatidylserine (PtdSer), gelatin nanoparticles, and DSPE-PEG_2000_ (1,2-distearoyl-sn-glycero-3-phosphoethanolamine-N-[amino(polyethylene glycol)-2000). First, gelatin nanoparticles were prepared as previously described with modifications 11. Briefly, gelatin type A was dissolved at 50 mg/mL in deionized (DI) water at 40 °C using continuous stirring on a temperature-regulated water bath. To the resulting solution an equal volume of acetone was added at 6.0 mL/min using a syringe pump while stirring at room temperature. Upon acetone addition, the stirring was stopped and the solution was left at room temperature for one minute to allow for layer separation. The upper layer containing the low molecular weight gelatin was then discarded and an equal amount of DI water was added to the lower layer and mixed by bath sonication. The resulting gelatin nanoparticles were analyzed by dynamic light scattering and the protein concentration was determined using Bradford protein assay with bovine serum albumin (BSA) as a standard.

Latex nanoparticles containing PtdSer, gadolinium lipids and fluorophore were prepared as previously described in great detail by our group 12. Next, a library of gelatin-latex nanoparticles was created using various concentrations of gelatin, DSPE-PEG_2000_ and latex (0.028±0.02 µm). The concentration of the components and physicochemical properties of some such nanoparticles is presented in Supplemental Table S2. To fuse gelatin and latex nanoparticles, the particles were mixed at various ratios in the presence or absence of 1 mg/mL liraglutide and bath-sonicated for 10 min at 200 W on ice. The particles were pelleted at 150,000 g x 20 min at 4^o^C (Thermo Sorvall MX 120 Plus micro-ultracentrifuge, S120-AT3 rotor) and resuspended in 25 mM HEPES buffered saline using bath sonication. The particles were further purified using Zeba Spin Desalting Columns, 40K MWCO (Thermo Fisher) and sterile-filtered using 0.2 µm polysulfone membrane. The “best” formulation was determined to incorporate 0.35 mg/mL of liraglutide, 0.29 mg/mL gelatin, and 2 mg/mL polystyrene latex.

The particle size was measured using dynamic light scattering (DLS) and transmission electron microscopy (TEM) after negative staining with uranyl acetate. FEI Technai G2 Spirit TEM operating at 80 kV was used to visualize GlpNP. The liraglutide concentration was determined by high performance liquid chromatography (HPLC) as described below. The nanoparticle purity (absence of non-incorporated gelatin and lipids) was confirmed using gel filtration chromatography on two Tosoh TSKgel columns connected in series. These were TSKgel SuperMultiporePW-H column and TSKgel G5000PXL both from Tosoh Bioscience (King of Prussia, PA). Shimadzu HPLC system was used as described below for liraglutide analysis. The analysis of the nanoparticles was performed after injection of 20 µL 0.1 mg/mL GlpNP or corresponding individual components of GlpNP and elution at 0.6 mL/min with 0.1 M ammonium formate in 10% acetonitrile in water at pH 6.5. Multimode detection was used to simultaneously detect gelatin (UV detector at 280 nm), fluorescent polystyrene (fluorescence detector at 480 nm excitation and 520 nm emission), and DSPE-PEG + other lipids, including Gd-lipids (SEDEX 90LT evaporative light scattering detector at 50^°^C and nitrogen gas pressure set at 3.5 bar).

## Results

### Glp1r is expressed in human and murine atherosclerotic plaque

Given the importance of myeloid cells in pathogenesis of atherosclerosis 13 and the previously reported expression of *Glp1r* in myeloid cells 14, we analyzed the levels of *Glp1r* mRNA transcripts in cultured primary cells derived from the bone marrow and peritoneum of Apoe^-/-^ mice. The expression of *Glp1r* was low or undetected in these cells (Supplementary material online, Figure S1A). The differentiation of these cells into macrophages and dendritic cells, or treatment with a panel of cytokines, oxidized LDL and apoptotic bodies to modulate their inflammatory/anti-inflammatory sub-phenotypes did not result in the induction of *Glp1r* expression (Supplementary material online, Figure S1A). Using a lineage tracking model of mice expressing Cre recombinase under the *Glp1r* promoter crossed with a ROSA26 eYFP reporter strain, low levels of GLP-1R expression were detected in splenic lymphoid and myeloid cell populations consistent with our observations in cells from Apoe^-/-^ mice (Supplementary material online, Figure S1B). However, *Glp1r* mRNA was notably elevated in whole atherosclerotic aortas of high-fat-fed (30 weeks on a Western diet) Apoe^-/-^ mice as compared to those from wild type, age-matched animals (Supplementary material online, Figure S1C).

**Figure 1:**
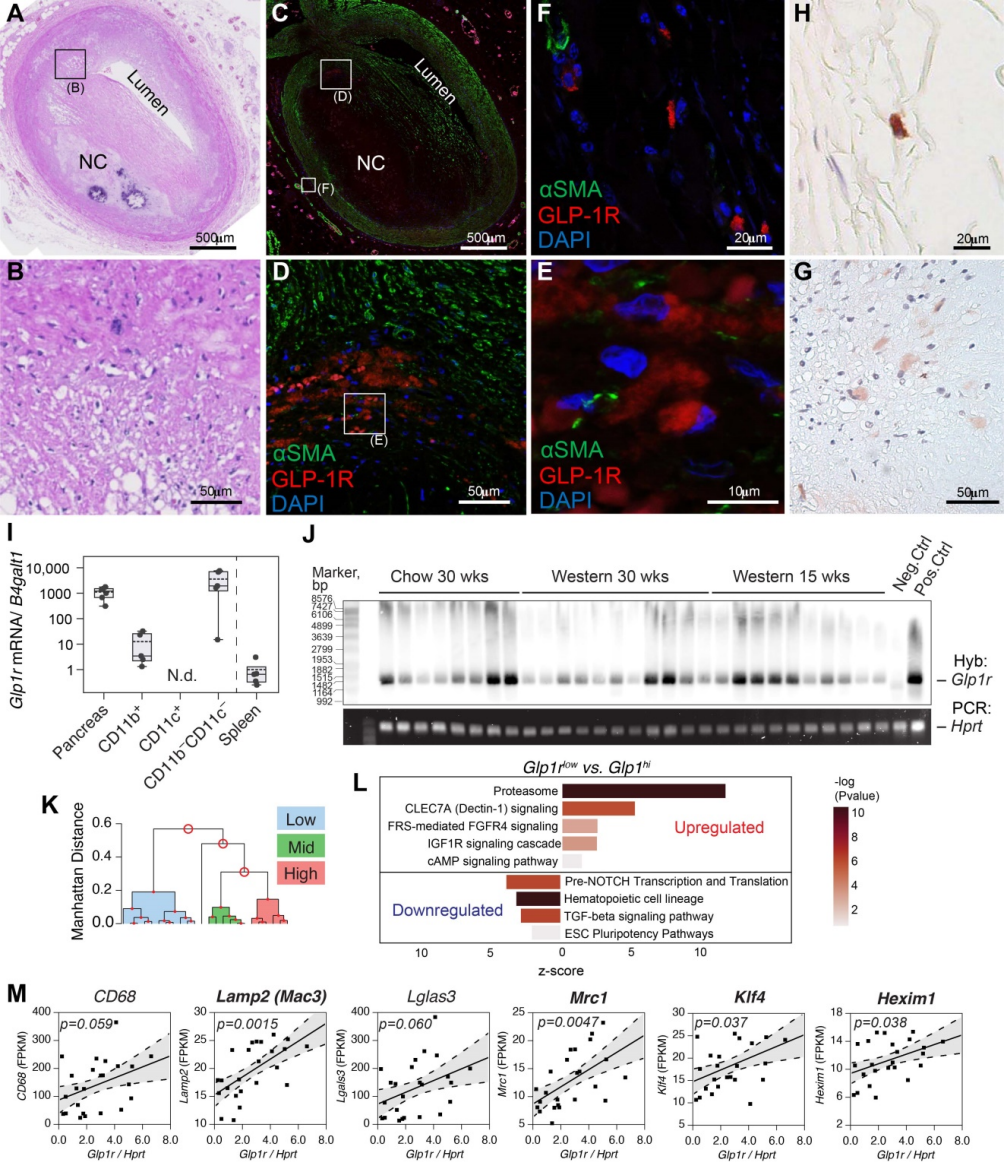
**GLP-1R is expressed in human and mouse atherosclerosis. A-H)** Human coronary artery pathology obtained from a 35 years old white male autopsy case with severe coronary mutivessel atherosclerotic disease and a history of prior myocardial infarction. **A**) Low power H&E staining section of atherosclerotic coronary artery (early fibroatheroma) in proximal LAD.** B**) Middle power H&E image of black rectangular field in A. **C**) IF staining image of αSMA (green) and GLP-1R (red) with DAPI nuclear staining (blue) which is adjacent to section A. (**D-E**) Middle power (**D**) and high power (**E**) image of white rectangular field in C and D. GLP1R (red) positive but αSMA (green) negative cells (likely macrophage) were observed in the field. **F**) High power image of adventitial lesion at white rectangular field in C. Multiple GLP1-R positive cells were observed. (**G-H**) IHC image of GLP1R positive cell (brown) in the fields of intra-plaque (G) and adventitia (H) which is obtained from adjacent fields of E and F. **I**) Atherosclerosis-resident macrophage-like (CD11b) and pan-negative fraction but not dendritic cell-like (CD11c) cells expressed *Glp1r* as determined via magnetic-bead sorting followed by real-time quantitative PCR. **J)** Full-length *Glp1r* mRNA transcripts in whole aortas from Apoe^-/-^ mice (fed as indicated) as analyzed by Southern blotting. *Hprt* served as a housekeeping control. Neg.ctrl - Glp1r^-/-^ lung, Pos.ctrl - Glp1r^+/+^ lung. **K**) Ward's hierarchical cluster analysis allowed for clustering of tissues expressing high, low and medium levels of *Glp1r*. **L**) mRNA from B was analyzed by RNA sequencing that demonstrated differentially regulated top canonical pathways comparing tissues expressing high vs. low levels of *Glp1r*. **M**) Results of the correlation analysis between *Glp1r* expression found in (J-L) and the levels of various myeloid and smooth muscle-related gene mRNA transcripts. Confidence belt is 85% and is shown in grey. Abbreviations: H&E - hematoxylin and eosin; LAD - left anterior descending artery; NC - necrotic core; DAPI - 4′,6-diamidino-2-phenylindole; N.d. - not detected.

To understand the relevance of these findings in human atherosclerosis, a library of human atherosclerotic plaques with varying stages of atherosclerosis was screened for their immunoreactivity to extensively validated 3F52 anti-GLP-1R monoclonal antibody 15. GLP-1R expression was noted in areas of the intima and adventitia in close proximity to CD68^+^ cells in plaque (Supplementary material online, **Figure** S1E). Similarly, in brachiocephalic arteries (BCA) of high-fat-fed Apoe^-/-^ mice (30 weeks on a Western diet) GLP-1R expression assessed with a validated monoclonal antibody mAb 7F38 demonstrated disperse staining with high intensity in artery media (Figure S1F). When examined across a range of atherosclerosis stages ([e.g diffuse intimal thickening, fatty infiltration, fatty streak, and pathological intimal thickening (PIT)], GLP-1R expression (**Figure 1A-H**), Supplementary material online, Figure S2-S4) appeared to be expressed predominantly in Type III lesions (PIT) which are known for the accumulation of intimal smooth cells with macrophage infiltration and lipid pool formation 16. GLP-1R expression in human specimens was noted almost exclusively in the intima (**Figure 1D, E**) with some expression in the adventitia (**Figure 1F**). Notably, most of the GLP-1R signal in the intimal lesions was observed within the large pool of ɑSMA^+^ cells, suggesting a potential smooth muscle cell origin (Supplementary material online, Figures S2-S3) 17. When co-stained with antibodies against alpha-smooth muscle actin (ɑSMA), GLP-1R^+^ cells localized within the intimal space, demonstrated spindle shape usually characteristic of vascular smooth muscle cells (VSMCs), and confined within a large lipid pool, however without complete colocalization. In contrast, mouse BCA sections co-stained with antibodies against ɑSMA, demonstrated medium-to-high level of co-localization (Figures S1F).

Primary mouse CD11b^+^ but not CD11c^+^ cells expressed *Glp1r* (**Figure 1I**), although the levels were low compared to pancreas. The expression levels in the negative fraction (CD11b^-^CD11c^-^) were high, although somewhat varied. Given these results, we set out to examine the expression of the full-length *Glp1r* mRNA in atherosclerosis. Using a high fidelity polymerase and a set of primers that yields a PCR product encompassing the majority of the GLP-1R open reading frame, we analyzed *Glp1r* expression in the whole aorta from thirty Apoe^-/-^ mice at different stages of atherosclerosis progression (**Figure 1J**). The expression was detected using Southern blotting with a hapten-tagged *Glp1r*-specific probe. A housekeeping gene hypoxanthine-guanine phosphoribosyltransferase (*Hprt*) that had stable expression across all samples examined was used for normalization (Supplementary material online, Figure S1D). When normalized against the level of *Hprt*,* Glp1r* expression levels in age-matched chow-fed and high-fat high-cholesterol-fed ApoE^-/-^ mice varied greatly, and were independent of the amount of time on the diet. To explore this variability and to gain insight into the pattern of the *Glp1r* expression, we performed bulk RNA sequencing (RNAseq) using the same RNA from which cDNA was generated for PCR amplification and Southern hybridization. We analyzed low vs. high *Glp1r* (*Glp1r*^low^ vs. *Glp1r*^hi^) expressing aortas (**Figure 1K**), including those from animals on a normal chow diet. Pathway analysis (**Figure 1L**) revealed that *Glp1r*^hi^ aortas had upregulated proteasome and CLEC7A signaling, indicative of heightened expression of pattern-recognition receptors (PRR) in myeloid cells and increased phagocytosis. TGFβ-signaling was downregulated, while TGFβ-signaling deactivating pathway fibroblast growth factor receptor/ receptor substrate 4 (FRS/FGFR4) was upregulated 18. TGFβ-signaling is central in vascular smooth muscle (VSMC) cell differentiation and proliferation 19, with its downregulation suggestive of de-differentiation of these cells to a macrophage-like population. These pathways have been extensively validated in VSMC-to-macrophage-like transition in atherosclerosis 17,18. There was a significant positive correlation of *Glp1r* expression with classic macrophage markers *Mac3* (also known as *Lamp2*), *Mrc1* (*CD206*) and others (**Figure 1M** and Supplementary Table S3), including transcription factor *Klf4*, which are all known to play a role in VSMC de-differentiation 17.

### Glp1r expression in smooth muscle cells as a function of de-differentiation

**Figure 2:**
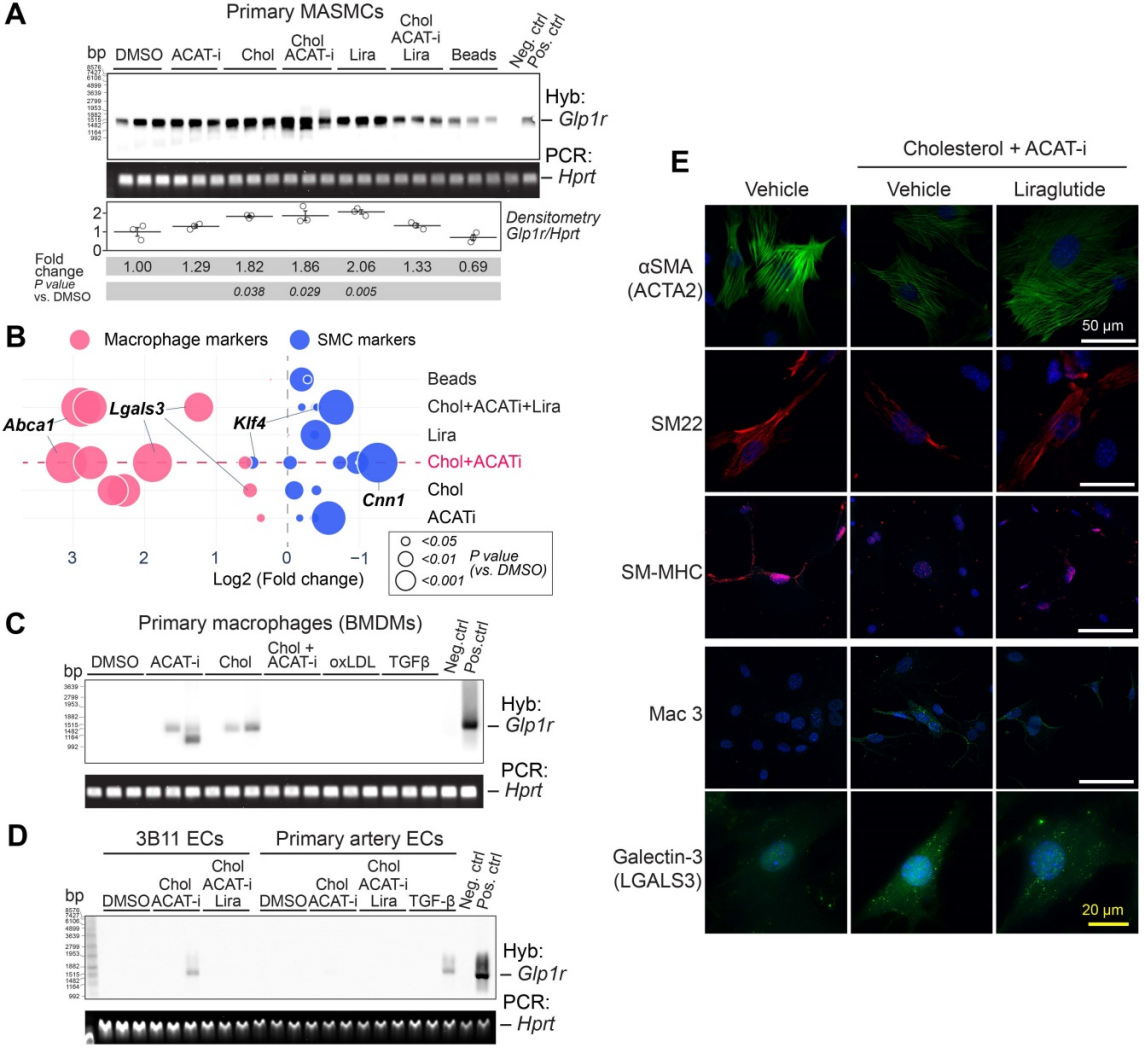
***Glp1r* is highly expressed in mouse aortic smooth muscle cells (MASMCs). A)** Southern blotting analysis of *Glp1r* expression in MASMCs from Apoe^-/-^ mice treated as indicated for 48 h. *Hprt* served as a housekeeping control. Neg.ctrl - Glp1r^-/-^ lung, Pos.ctrl - Glp1r^+/+^ lung. **B**) Markers characteristic of VSMCs (blue) or macrophages (red) as examined by qPCR. Fold change (FC) vs. DMSO control is shown as a bubble chart, with the bubbles sized by their p value, as analyzed by ANOVA with the Tukey *post hoc* test. The complete list of markers is presented in the Supplementary Figure 6A and the detailed statistical analysis is presented in Table S4. **C**) *Glp1r* expression in BMDMs treated as indicated and analyzed by Southern blotting as in (A). **D**) *Glp1r* expression in the murine endothelial cell (EC) line 3B11 and in the primary ECs isolated from carotid artery, treated as indicated (and in the Supplementary material online, methods) and analyzed by Southern blotting as in (A, B). **E)** Immunofluorescence staining of VSMC and macrophage markers (red and green respectively) in MASMCs subjected to the treatments as indicated. Nuclei were counterstained with Hoescht (blue).

In order to examine the role of SMC-to-macrophage transition on *Glp1r* expression, we cultured primary mouse aortic smooth muscle cells (MASMCs) under various cholesterol-loading conditions. In agreement with prior work by others 19, appreciable cholesterol loading was achieved only with free cholesterol (complexed with cyclodextrin), but not with modified low density lipoproteins or other commonly used conditions (Supplementary material online, Figure S5A). Acetyl-CoA acetyltransferase inhibitor CP-113818 (ACAT-i) was used to maintain high intracellular levels of free cholesterol in MASMCs for 48 h. The phenotype of “transitioned” smooth-muscle cells to a macrophage phenotype was confirmed in cholesterol-loaded-MASMCs by RNAseq and was in agreement with the previously reported expression signature (Supplementary material online, Figure S5B) 20. Southern blotting analysis (**Figure 2A**) demonstrated significant upregulation of the *Glp1r* expression, as compared to vehicle, cholesterol or ACAT-i only controls in the transitioned cells. Co-incubation with 100 nM GLP-1R agonist liraglutide (Lira) appeared to attenuate the *Glp1r* expression in these cells in response to cholesterol loading, suggesting potential receptor downregulation. The changes in *Glp1r* expression in response to cholesterol positively correlated with loss of SMC markers and gain of macrophage-like markers, including *Abca1*, *Lgals3* and *Lamp2* (*Mac3).* Notably, the SMC dedifferentiation marker *Klf4* was highly upregulated (**Figure 2B**) 17. Lira treatment partially rescued the SMC phenotype, most significantly manifested in *Abca*1 and *Lgals3* downregulation (**Figure 2B** and Supplementary material online, **Figure** S6A).

Importantly, cultured BMDMs, popular endothelial cell line 3B11 and primary aortic endothelial cells (ECs) displayed no or very low *Glp1r* expression in response to either cholesterol or TGFβ (**Figure 2C,D**). TGFβ was previously speculated to cause macrophage-to-SMC transdifferentiation 21 and endothelial-to-mesenchymal transition (EndoMT) 22. Nevertheless, neither of these atherosclerosis-relevant *in vitro* conditions were able to induce *Glp1r* expression, even when ECs were expressing high levels of smooth muscle markers *Acta2* and *Cnn1* (Supplementary material online, Figure S6B). Importantly, GLP-1R was highly expressed on the protein level in MASMCs, as demonstrated by immunofluorescence experiments using mAb 7F38 (Supplementary material online, **Figure** S6C).

In cholesterol-loaded MASMCs, Lira restored the expression of ɑSMA (encoded by *Acta2*), SM22 (also known as transgelin and encoded by *TAGLN*), smooth muscle myosin heavy chain (SM-MHC, encoded by *Myh11*), and downregulated macrophage-like markers such as Mac3 (encoded by *Lamp2*), and Galectin-3 (encoded by *Lgals3*) (**Figure 2E**).

Collectively, these observations suggest that *Glp1r* expression occurs in atherosclerosis, but primarily in vascular smooth muscle cells. Myeloid cells (macrophages, monocytes) or endothelial cells are unlikely to be the source of the cells expressing* Glp1r* in atherosclerosis. This was further confirmed using mAb 7F38 and co-staining in Apoe^-/-^ BCA sections with macrophage (CD68) and endothelial cell (VE-Cadherin) markers (Supplementary material online, **Figure** S7). The data above provided a sound rationale for the delivery of a GLP-1R agonist Lira as an intervention in atherosclerosis with the goal of targeting cholesterol-loaded smooth muscle cells to effectively prevent smooth muscle-to-macrophage transition.

### Characterization of proteolysis resistant, MMP-activatable GlpNP

**Figure 3:**
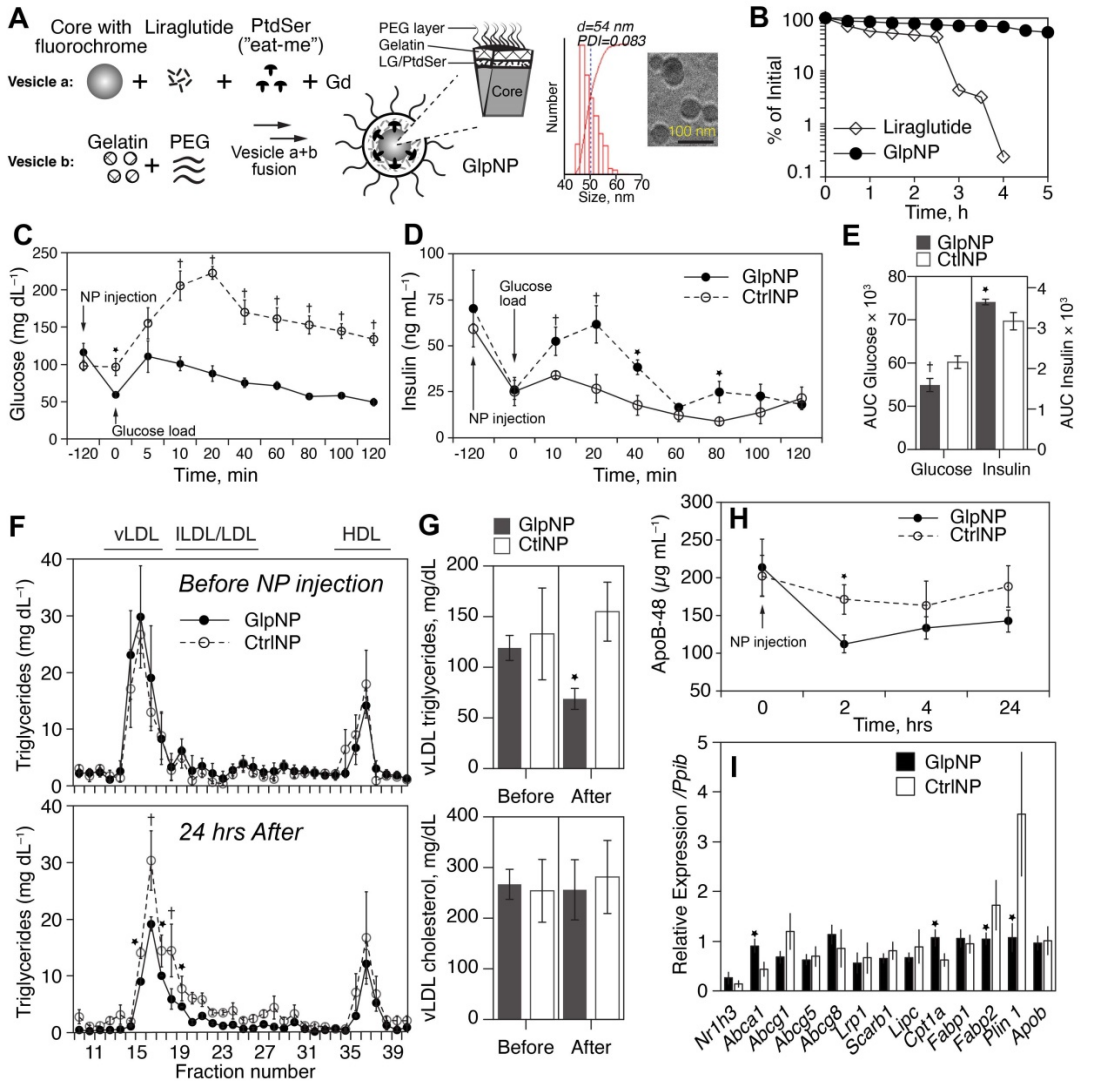
**GlpNP carrying the GLP-1R agonist liraglutide are bioactive and resistant to proteolysis. A)** GlpNP were synthesized from a fluorescent core, Lira, gadolinium-DTPA and PEG lipids. Inset shows DLS and cryo-TEM characterization of GlpNP. **B)** GlpNP are resistant to proteolysis by neutral endopeptidase (NEP) in cell-free assays as determined by HPLC. Average of two independent experiments is shown. **C)** GlpNP but not CtrlNP improved oral glucose tolerance (OGTT) after a single I.V. injection 2 h before the glucose challenge and followed by a glucose load. **D)** GlpNP promoted insulin release in the same set of experiments. **E)** Area under the curve (AUC) quantification derived from OGTT and insulin release in C and D, respectively. **F**) Plasma from fasting Apoe^-/-^ mice injected with NPs was subjected to fast protein liquid chromatography (FPLC) analysis at baseline and 24 hrs after injection, and triglyceride levels were measured in the eluted fractions. **G)** Area under the curve analysis of FPLC chromatograms quantified the levels of cholesterol and triglycerides. **H)** ApoB-48 concentrations in plasma measured over time after NP administration.** I)** qPCR analysis of the lipid metabolism-related genes in the liver of animals treated with NPs for 24 h. *p<0.05, †p<0.01* via* two-way ANOVA, Tukey's post-hoc correction, n=5-7 animals (C, D) and pairwise T-test with Holm post-hoc correction (F, G, H). n=3-4 animals.

We next evaluated the impact of a novel plaque-targeted nano-GLP1 agonist at very low doses to allow decoupling of systemic effects in an effort to understand the impact of direct plaque delivery on atherosclerosis. Given recent data indicating that the majority of foam cells 23 and inflammatory cells in plaque could originate from SMCs and our preliminary data suggesting *Glp1r* expression in de-differentiated SMCs that express macrophage markers, we designed and synthesized multilayered nanoparticles (NPs) decorated with phosphatidylserine (PtdSer) “eat-me” signals that allow for delivery and uptake 24. These engineered NPs were coated with polyethylene glycol (PEG) to increase their lifespan in circulation, and equipped with an outer gelatin coat, allowing their selective cleavage in the presence of gelatinases within plaque (**Figure 3A** and Supplementary material online, Figure S8A) 25. The inclusion of the GLP-1R agonist Lira between the particle's core and gelatin layer allowed for “context specific” release upon cleavage by matrix metalloproteinases (gelatinases) present in plaque. A scheme depicting the proposed mechanism of action of these NPs, termed GlpNP, is presented in Supplementary material online, Figure S8A. Size and drug loading characteristics were determined for various GlpNP formulations, and the optimal formulation was selected (Supplementary Table S2). Two controls were used, one containing PtdSer (CtrlNP) but without Lira, and another lacking both PtdSer and Lira (PtdSer^neg^CtrlNP). We tested if GlpNP display PtdSer in the presence of gelatinases as described in Supplementary material online, Figures S8B-D.

DPP-4 and neutral endopeptidase (NEP, neprilysin) are the two most important enzymes that catalyze GLP-1 (and its clinically-used analogs) proteolytic inactivation *in vivo 26*. Given the fact that GlpNP particles were “protected” by both PEG and the gelatin shell, we reasoned that GlpNP would be especially resistant to enzyme-driven degradation in plasma 27. Indeed, **Figure 3B** and Supplementary material online, Figure S9 show high-performance liquid chromatography (HPLC) analysis of incubation of GlpNP with NEP that demonstrated resistance to proteolytic hydrolysis, when compared to that of Lira alone. Physicochemical characterization of GlpNP in the absence of the enzymes confirmed that the particles were intact and had protein, phospholipid and polystyrene signatures, as determined by Fourier-transformed infrared spectroscopy and size exclusion chromatography (Supplementary material online, Figures S10A,B). GlpNP did not induce significant cytotoxicity in MASMCs and macrophages over the range of concentrations 0.1-100 µg/mL (Supplementary material online, Figures S10C). However, in the presence of gelatinases GlpNP rapidly released Lira in a time-dependent manner (Supplementary material online, Figures S11A).

Lira retained *in vitro* biological activity when incorporated within the NPs (Supplementary Figure 11B) manifesting in prototypical cyclic AMP (cAMP) release from RINm5F rat pancreatic islet insulinoma cells incubated with GlpNP or relevant controls. Importantly, *in vivo* biological activity of GlpNP was assessed in Apoe^-/-^ mice. A single intravenous injection of 30 µg/kg GlpNP (based on Lira concentration in GlpNP) but not CtrlNP in high-fat-fed mice (20 weeks on a Western diet), resulted in potent glucose lowering and insulinotropic effects following an oral glucose challenge (**Figure 3C-E**).

Consistent with prior observations, we noted reductions in fasting levels of triglyceride rich lipoproteins in the GlpNP-, but not in CtrlNP-injected mice (**Figure 3F, G**) 28. Apolipoprotein B-48 (ApoB-48) was also reduced after GlpNP injection (**Figure 3H**). Total plasma cholesterol or low- and high-density lipoprotein cholesterol (LDL and HDL) did not change with GlpNP treatment (**Figure 3F, G** and Supplementary material online, Figure S12A). The analysis of common lipid-regulating genes in liver homogenates (**Figure 3I**) demonstrated significant upregulation of mRNA for *Abca1* (cholesterol transport), downregulation of *Fabp2* (triglyceride-rich lipoprotein synthesis), and downregulation of *Plin1* (marker of mature lipid droplets). These data seemed to suggest reduced lipid accumulation and enhanced cholesterol efflux in response to GlpNP. These effects were recapitulated in human primary aortic smooth muscle cells (HASMC) incubated with acetyl-LDL in the presence of GlpNP or Lira, which contained fewer lipid droplets in the GlpNP group, but not in the two control groups (Supplementary material online, Figure S12B). HASMC, which were cholesterol-loaded with acetyl-LDL plus BODIPY-cholesterol followed by HDL-mediated cholesterol efflux, demonstrated higher efflux in GlpNP-treated cells vs. CtrlNP (without Lira) and these effects were dependent on cAMP activation and blocked by a large excess of GLP-1R antagonist exendin (9-39) (Supplementary material online, Figure S12C).

### Acute GlpNP administration targets atherosclerotic plaque allowing for theranostic application

**Figure 4:**
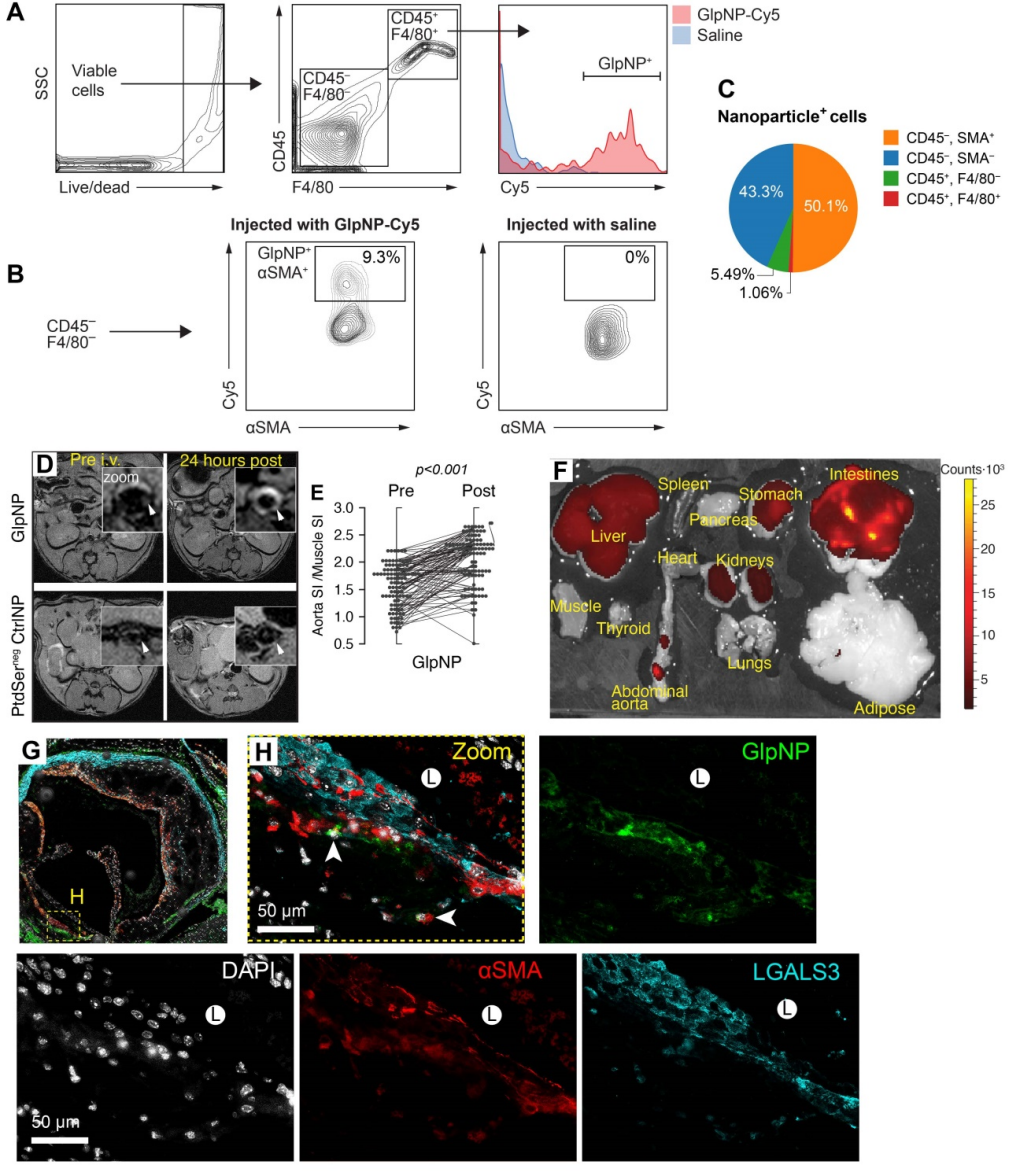
**GlpNP target myeloid and SMCs in atherosclerosis. A, B)** GlpNP preferentially target myeloid and smooth muscle cells in atherosclerosis as shown by flow cytometry. Apoe-/- mice were bolus-injected with saline or Cy-labeled GlpNP and the aortas were extracted 24 h later. Single-cell suspensions from enzymatically-digested whole aortas were stained with antibodies against CD45, F4/80 and αSMA. **C**) Myeloid (CD45^+^) and non-myeloid (CD45^-^) cells in atherosclerotic plaque demonstrated GlpNP positivity, with the highest expression seen in αSMA-positive cells, as shown in the pie diagram demonstrating total GlpNP positivity among indicated cell types. **D**) MRI of atherosclerotic plaque after administration of gadolinium-loaded GlpNP or PtdSer^neg^CtrlNP as indicated. **E**) Signal-to-noise measurements from (D) represented as signal intensities (SI) in aorta vs. SI in muscle. Significance was determined by repeated measures ANOVA. **F**) Whole-organ fluorescence imaging 24 h after GlpNP administration. Red-yellow signal is indicative of GlpNP accumulation in the given organ as annotated. **G, H**) Localization of GlpNP in aortic root tissue sections as seen by fluorescence microscopy. GlpNP fluorescence (pseudo-colored green, arrows) was observed in close proximity with the cells expressing macrophage (LGALS3, cyan) and smooth muscle (αSMA, red) markers. Nuclei (blue) were counterstained with DAPI. L - indicates plaque lumen.

Flow cytometry analysis demonstrated that both GlpNP and CtrlNP were engulfed by blood monocytes and accumulated within CD11b^+^CD11c^+^ cells (Supplementary material online, Figure S13A, B) 29. However, ɑSMA^+^ cells were the locus of GlpNP accumulation in atherosclerotic plaque, while CD45^+^F4/80^+^ macrophages were targeted by GlpNP to a slightly lesser degree (50.1 vs. 43.3% of total NP+ cells, respectively), as shown in **Figures 4A-C** and Supplementary material online, Figures S13C, D.

Plasma GlpNP levels were detectable by their intrinsic fluorescence after a bolus intravenous injection (30 µg/kg). Their persistence in circulation was observed for up to 7 h (Supplementary material online, Figure S14A).

To further demonstrate targeting and accumulation of GlpNP in atherosclerosis, we performed magnetic resonance imaging (MRI) of plaque in Apoe^-/-^ mice with advanced atherosclerosis (30 weeks on a Western diet). The animals underwent treatment with a single dose of GlpNP, CtrlNP and PtdSer^neg^CtrlNP, all containing 0.05 mmol/kg of gadolinium (Gd) lipids as a T1-weighted MR contrast agent. MR imaging demonstrated that GlpNP significantly accumulated in atherosclerotic plaque as compared to NPs without PtdSer, refered to as PtdSer^neg^CtrlNP (**Figure 4D, E**). Notably, CtrlNP containing PtdSer, but not Lira, also accumulated within atherosclerotic plaque (Supplementary material online, Figure S14B), indicating that particle retention in plaque was not dependent on Lira-GLP-1R interaction. Furthermore, when gelatin coating was omitted in the synthesis of Lira-containing NPs (Gelatin^neg^GlpNP), prominent accumulation in tissues known to express *Glp1r* (pancreas, mesenteric adipose, lung) 4 was noted (Supplementary material online, Figure S14C). This, however, did not occur when Gelatin^neg^GlpNP was co-administered with a 10-fold excess of free Lira (300 µg/kg). This is suggestive that the gelatin coating in GlpNP protected from agonist-receptor binding in the off-target tissues allowing for site-selective accumulation in plaque. GlpNP biodistribution studies 24 h post injection detected GlpNP in abdominal aorta and off-target organs such as liver and kidneys (**Figure 4F**). Deposition of NPs in atherosclerosis was confirmed by immunofluorescence in aortic root sections (**Figure 4G,H**) and BCA (Supplementary material online, Figure S15).

### Chronic delivery of GlpNP containing a very low dose of liraglutide alleviates plaque pathology

**Figure 5:**
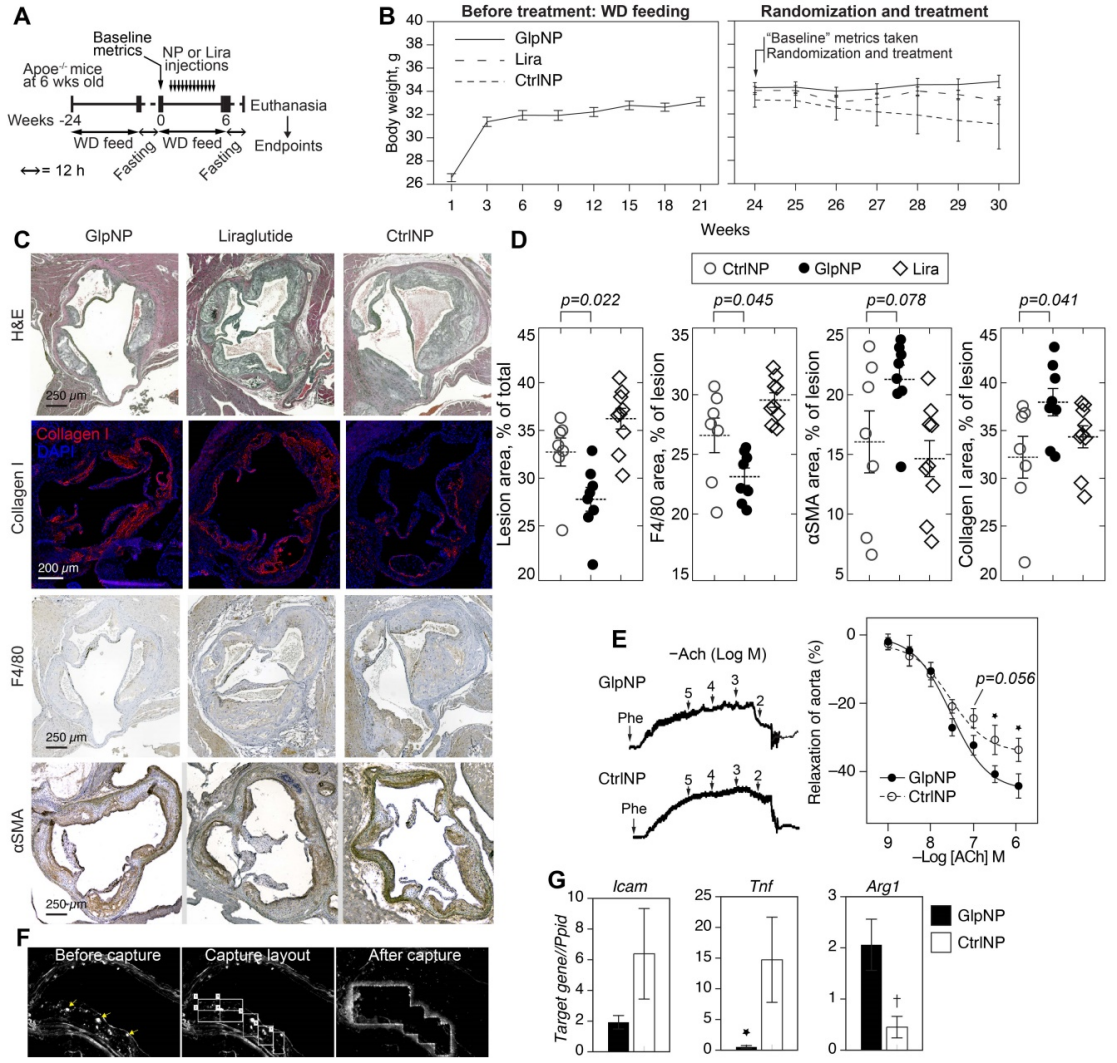
** GlpNP reduced plaque burden without changes in body weight. A)** Apoe^-/-^ mice were fed Western diet (WD) for 24 weeks to induce atherosclerosis. Animals were then fasted as indicated and baseline metrics were taken as described in Methods and the text. Mice were continued on WD and received GlpNP, CtrlNP or Lira (1 μg/kg based on Lira or 100 µg of particles in CtrlNP) I.V. for 6 weeks twice weekly.** B)** Body weight measurements over time before and after randomization. **C)** Morphometric assessment of plaque burden with representative sections of aortic root after indicated staining. Sections were stained with Hematoxylin Eosin (H&E, general tissue morphology), Collagen I, F4/80 (macrophage-like cells), alpha smooth muscle actin (ɑSMA). **D)** Quantification of IHC staining indicated 15% reduction in aortic sinus lesion area and 13% decrease in macrophage content, while Collagen I expression increased by 18% (GlpNP vs. Lira) via pairwise T-test with Holm post-hoc correction. n= 7-9/group. **E)** Concentration-response curves recorded in the presence of acetylcholine (Ach) of aortic rings from animals injected with NPs. Pre-contraction was induced with 10^-5^ M phenylephrine (Phe). **F**) Aortic sections from mice injected with NPs were subjected to laser capture microdissection, after which targeted cells were identified by characteristic NP fluorescence (arrows).** G)** Captured cells were analyzed according to mRNA expression of *Icam*, *Tnf* and *Arg1*. *p<0.05, †p<0.01 vs CtrlNP via pairwise T-test with Holm correction in G and ANOVA with Tukey post-hoc test in E. n= 3-4/group.

We next tested the ability of GlpNP containing a sub-systemic dose to Lira to modify the progression and stability of established atherosclerotic lesions in chronic intervention experiments. The sub-systemic dose of Lira in injected GlpNP was deliberately selected to be substantially lower (1 μg/kg) than previously described doses of GLP-1R agonist that had effects on atherosclerosis and weight loss 30. Nineteen Apoe^-/-^ mice were maintained on a Western diet for 24 weeks, after which animals were randomized into different groups (GlpNP, CtrNP and Lira) and were assessed for their body weight, glycemic metrics and plasma lipids (**Figure 5A**, **Table 1**). These measurements were referred to as “baseline”. Animals were then started on intravenous injections of GlpNP, CtrNP or Lira twice weekly for six weeks while continuing with the high-fat diet regimen. No significant body weight change at week 30 was observed between treatment groups, including Lira alone (**Figure 5B**). GlpNP reduced the atherosclerosis burden (**Figure 5C,D**) and macrophage content (F4/80^+^ cells). Necrotic core was also reduced (not shown), although this did not reach significance in GlpNP vs. Lira groups. Notably, we observed a trend of an increase in ɑSMA, and statistically significant increase in collagen I, both of which are plaque stabilization markers 17.

A comprehensive assessment of metabolic parameters at the end of the experiment (**Table 1**) demonstrated no significant changes in glycemia and insulin, lipids or lipoproteins across all treatment groups, except for the levels of vLDL and total triglycerides, which were reduced in the GlpNP group. Liquid chromatography mass-spectrometry (LCMS) quantification revealed a significant decrease in total and free cholesterol in whole atherosclerotic aortas of GlpNP-treated animals as compared to Lira- or CtrNP-treated mice (**Table 1**).

Given that GLP-1 has previously been associated with improved vascular function 31, and that smooth muscle cells are likely to be targeted by GlpNP in plaque, we tested the effects of GlpNP on vasorelaxation (**Figure 5E**). Our results show only a trend towards an increase in aortic relaxation in GlpNP-treated mice at acetylcholine (ACh) concentrations lower than 100 nM, which is indicative of only somewhat improved endothelial function 32.

Gene expression analysis in plaque GlpNP^+^ (vs. CtrNP^+^) cells isolated using laser capture microdissection showed that GlpNP-targeted cells exhibited a lower inflammatory status, manifested by the decrease in *Tnf* and *Icam1* expression with a concomitant increase in *Arg1*, a marker of alternative macrophage activation (**Figure 5F, G**). Supplementary material online, Figure S16 details additional genes tested which demonstrate a downregulation in the expression of inflammatory genes. Overall, our data indicate that GlpNP can selectively target atherosclerotic plaque, where they appear to accumulate in VSMC-like cells and favorably modulate atherosclerosis.

## Discussion

In this study, we demonstrate the feasibility of a degradation-resistant GlpNP at weight-neutral ultra-low doses to target atherosclerotic plaque. We further demonstrate *Glp1r* expression in atherosclerosis and plaque-associated cells, which functionally resemble macrophages but appear to be of smooth muscle origin. The expression of *Glp1r* in immune cells and in the cardiovascular system has been a topic of considerable debate 32. Interpretation of studies has been further complicated by the lack of commercial availability of antibodies with specificity for murine GLP-1R 5,6. In many studies, the demonstration of a physiological or therapeutic effect of GLP-1 or commercially available GLP-1R agonists on cells, organs or* in vivo* has been considered proof of *Glp1r* signaling 4. Recent studies have started to support the idea that GLP-1 may exert its effects in the absence of *Glp1r* in the tissue, via intermediary molecules such as atrial natriuretic peptide, particularly in the heart, which has low level of *Glp1r* expression 9. Other reports have suggested that biologically active degradation products of GLP-1, such as GLP-1(9-36) and GLP-1(28-36), either directly or via an as yet to be described receptor may mediate the effects of GLP-1R 33.

Using a variety of approaches including lineage tracing and Southern blotting, we confirm that *Glp1r* was expressed at very low levels* in vivo* in myeloid CD11b^+^ cells, in cultured macrophages, other immune cells and endothelial cells 5,6. These data seem to suggest that myeloid cells are unlikely to be a major source of *Glp1r* in atherosclerosis. We therefore posited that macrophage-like cells could potentially represent de-differentiated vascular smooth muscle cells. Recent data seem to strongly suggest that the vast majority of macrophage and foam cells in atherosclerosis may indeed have smooth muscle cell origins 17,23. Indeed, cultured mouse aortic smooth muscle cells expressed *Glp1r* transcript, which increased in response to cholesterol loading, a well-known stimulus to induce SMC dedifferentiation and SMC-to-macrophage transition. Further, *Glp1r* expression was positively correlated with hallmarks of dedifferentiation (such as increased *Klf4*) 17. loss of SMC markers and gain of macrophage-like markers (*Abca1*, *Lgals3* and *Mac3*) 17. In contrast, the induction of endothelial-to-mesenchymal transition upon treatment of ECs with TGFβ was not sufficient to induce *Glp1r* expression in primary ECs that lacked noticeable baseline *Glp1r* expression*. Glp1r* expression in experimental atherosclerotic plaque was highly variable but aortas from Apoe^-/-^ mice expressing high levels of *Glp1r* demonstrated downregulated TGFβ-signaling, important in VSMC dedifferentiation. The upregulation of CLEC-1 (dectin-1), FGGR1, IGF1R and cAMP signaling is consistent with a “macrophage-like” phenotype. In line with our mouse studies, human Type III atherosclerotic lesions (PIT) expressed GLP-1R in spindle shaped cells resembling VSMC's in the midst of CD68^+^ and ɑSMA^+^ cells.

*In vivo* targeting of the GlpNP system was achieved with PtdSer, an “eat-me” signal coated with gelatin and PEGylated, designed to be resistant to plasma proteases and subject to degradation within the milieu of the plaque by gelatinases in plaque while enabling prolonged plasma residence time. We used NPs in the size range of 54±12 nm that may not readily cross the blood-brain barrier, which is consistent with the effects noted in our study including lack of weight loss. Lira release was shielded by the gelatin layer and improved its resistance to proteolysis by DPP-4 and NEP and unintended GLP-1R ligation. Interestingly, GlpNP were actively engulfed by monocytes with ɑSMA^+^ cells being the locus of GlpNP accumulation in atherosclerotic plaque, while CD45^+^F4/80^+^ macrophages were targeted by GlpNP to a lesser degree. MR imaging demonstrated significant accumulation in atherosclerotic plaque through a PtdSer-dependent mechanism.

Notably, the GlpNP system may be one of the very few described that draws therapeutic benefits from unintended, “off-target” accumulation. A portion of the injected GlpNP accumulated in the liver, which is not surprising, given the fact that most nanoparticles are known to deposit in the reticuloendothelial system despite any targeting or activatable features 34. Although many nanoparticle technologies are motivated by the promise of reducing side effects through site-specific delivery, many still suffer from unintended liver accumulation and may contribute to hepatic dysfunction or impairment of liver metabolism. For example, Fisher E.A. and colleagues demonstrate potent plaque reduction with NPs carrying liver X receptor agonist (NP-LXR), however, the authors also note triglyceride levels increased by 20% in NP-LXR group vs. NP without LXR agonist 35. In contrast, our GlpNP technology enables synergistic effects on liver and plaque, manifested in the reduction of triglycerides and plaque pathology. We did not specifically test the liver toxicity of GlpNP, as it is beyond the scope of this manuscript, however, we believe that this next generation of non-toxic, biodegradable and biocompatible GlpNP may benefit liver metabolism and insulin sensitivity, especially if administered at low doses as demonstrated by our work.

It is likely that the low dose used in our study was the reason why only a very modest effect on vascular function was observed: ACh-mediated relaxation was statistically significant only at high ACh concentrations (Figure 5E). The response to ACh is mainly endothelium-dependent 32, and thus the lack of the robust effect may also be explained by the fact that GlpNP had restricted accumulation in plaque intima, rather than in the endothelium.

Clinically used GLP-1R agonists are not devoid of side effects. For example, when delivered systemically, they cause gastrointestinal distress in ~25% of individuals. Some adverse effects may also arise through sympathetic nervous system (SNS) activation. However, as medium- and large-sized NPs (≥50 nm) such as GlpNP, do not cross the blood-brain barrier, the off-target effects seen with systemic delivery of GLP-1R agonists may be averted.

We specifically selected a weight-neutral ultra-low dose (1 µg/kg) for chronic delivery. This contrasts with clinical doses of 10-30 µg/kg of liraglutide (referred to as “high dose” in this paper), which is typically associated with weight loss, improved glycemic control and reduction in blood pressure 36. Using such a dose allowed us to dissociate actions associated with lowered body weight and potentially additional hemodynamic and systemic anti-inflammatory effects.

It is worth noting that the anti-inflammatory effects seen in previously published works remain controversial. Studies that investigated the effects of GLP-1R agonism in atherosclerosis in the context of monocyte adhesion 37, foam cell formation 38, and macrophage M1/M2 phenotypes 39 all used high doses of GLP-1R agonists (100-300 µg/kg) with either daily injections or continuous osmotic pump infusions; with one study employing adeno-associated virus overexpression of the GLP-1 and its split products 40. Unfortunately, many such studies, including a recent article describing GLP-1 effects in vascular endothelium 41, have not convincingly demonstrated the expression of functional *Glp1r* (encoding the open-reading frame of GLP-1R protein) thus making conclusions of these studies hard to interpret. The use of non-validated anti-GLP-1R antibodies, which plagued the antibody vendor market in recent years, was a common occurrence in many studies.

Nevertheless, to the best of our knowledge, our study is the only investigation to date that profiled multiple cell types found in atherosclerosis *in vitro* and *in vivo* using lineage tracing and Southern blotting in mice and staining in mouse and human plaques.

## Conclusions

In this study we demonstrate through carefully performed* in vitro* and *in vivo* studies including lineage tracing that *Glp1r* expression occurs at insignificant levels in hematopoietic cells but upregulated in de-differentiated VSMCs in response to SMC-to-macrophage transition. *Glp1r* expression was noted in human Type III atherosclerosis; known for a high number of de-differentiated VSMCs. Importantly, delivery of a non-weight loss inducing dose of GlpNP demonstrated important trends in metrics of plaque stabilization and reduced plaque cholesterol. These results, in conjunction with targeting of vascular smooth muscle cells and enhanced cholesterol efflux support the hypothesis of plaque pacification in response to targeted GLP-1R delivery. Our study has important implications on understanding of GLP-1 biology in atherosclerosis and highlights the possibility of human translation of plaque-targeted GLP-1R agonists. We describe a new technology of gelatinase-activatable nanoparticles. We believe that such a technology may find future use in multiple diseases where MMP2/MMP9 gelatinases are overexpressed and play a pathogenic role (including cancer). Thus, our paper is an important proof-of-concept study that demonstrates the potential of such a technology. The use of GLP-1R agonist liraglutide as a model cargo provided compelling evidence of effectiveness of GlpNP technology.

## Limitations

We acknowledge that we have not provided conclusive evidence that the effects of our targeting strategy are exclusively mediated by vascular expression of *Glp1r.* It is possible that degradation products of liraglutide via NEP may still mediate effects 33. There were technical limitations of this study, such as that we were not able to co-stain GLP1R, ɑSMA and CD68 in the same human atherosclerosis specimens. We also acknowledge that our results may also involve other poorly understood indirect neural or gastrointestinal pathways. The lack of significant weight loss, systemic delivery avoiding the gut and nanoparticle size that may not penetrate the blood brain barrier, however lowers the likelihood of these as significant contributors.

## Supplementary Material

Supplementary methods, figures and tables.

## Figures and Tables

**Table 1:** Metabolic parameters after six weeks of treatment with GlpNP, liraglutide or CtrlNPs.

	GlpNP		Liraglutide		CtrlNP	
	Baseline	Treatment	Baseline	Treatment	Baseline	Treatment
*Body weight and glycemia metrics*						
Body weight, g	34.26 ± 1.30	34.79 ± 1.54	34.12 ± 1.05	33.36 ± 0.81**^†^**	33.35 ± 1.93	31.64 ± 5.72
Fasting insulin, ng/mL	1.4 ± 0.2	1.5 ± 0.2	1.3 ± 0.1	1.8 ± 0.3	1.3 ± 0.3	1.1 ± 0.2
Fasting glucose, mg/mL	146 ± 16	150 ± 21	140 ± 29	138 ± 18	153 ± 17	178 ± 26
*Plasma cholesterol, lipids and lipoproteins*						
Fasting total cholesterol, mg/dL	788 ± 21	802 ± 21	792 ± 23	811 ± 25	791 ± 27	842 ± 30
Fasting total triglycerides, mg/dL	84 ± 18	48 ± 11^*^	79 ± 12	86 ± 12	83 ± 8	92 ± 18
HDL cholesterol, mg/dL (FPLC)	32 ± 3	23 ± 7	34 ± 12	35 ± 9	36 ± 7	34 ± 3
LDL cholesterol, mg/dL (FPLC)	217 ± 23	226 ± 35	215 ± 28	217 ± 24	215 ± 37	234 ± 19
vLDL cholesterol, mg/dL (FPLC)	332 ± 19	359 ± 25	334 ± 26	353 ± 29	341 ± 21	289 ± 33
HDL triglycerides, mg/dL (FPLC)	12 ± 2	15 ± 3	23 ± 12	21 ± 4	18 ± 12	23 ± 13
LDL triglycerides, mg/dL (FPLC)	34 ± 1	21 ± 3	35 ± 2	36 ± 4	31 ± 4	37 ± 3
vLDL triglycerides, mg/dL (FPLC)	92 ± 12	57 ± 10^*^	101 ± 19	123 ± 23	99 ± 15	133 ± 23
*Liver lipids*						
Liver cholesterol, µg/mg		39.2 ± 5.1		34.2 ± 9.1		35.6 ± 2.4
Liver triglycerides, µg/mg		282.3 ± 28.3		290.3 ± 29.6		289.2 ± 26.5
*Atheosclerotic plaque cholesterol*						
Plaque total cholesterol, nmoles/mg (LCMS)		**35.23 ± 2.89^§^**		49.43 ± 9.12		43.02 ± 3.76
Plaque free cholesterol, nmoles/mg (LCMS)		**28.32 ± 4.24^§,^** ^¶^		36.67 ± 2.54		40.12 ± 3.87
Plaque esterified cholesterol, nmoles/mg (calculated)		16.92 ± 2.12		12.76 ± 4.31		13.1 ± 3.45

Data presented are mean ± stdev. Group animal numbers were as follows: GlpNP n=8, Liraglutide n=9, CtrlNP n=6. ^†^P = 0.06; ^*^P < 0.01 vs. baseline; ^§^P < 0.05 vs. Liraglutide; ^¶^P = 0.0047 vs. CtrlNP. FPLC - fast performance liquid chromatography; LCMS - liquid chromatography mass-spectrometry.

## Abbreviations

GLP-1: glucagon like peptide-1
GLP-1R: glucagon like peptide-1 receptor
Apoe: Apolipoprotein E
NPs: nanoparticles
MRI: magnetic resonance imaging
cAMP: adenosine 3',5'-cyclic monophosphate
LDL: low density lipoprotein
ROSA26: reverse orientation splice acceptor 26
eYFP: enhanced yellow fluorescent protein
BCA: brachiocephalic artery
ɑSMA: smooth muscle actin, alpha
PCR: polymerase chain reaction
Hprt: hypoxanthine-guanine phosphoribosyltransferase
CLEC7: C-type lectin domain family 7
TGFβ: transforming growth factor beta
Klf4: kruppel-like factor 4
Abca1: ATP-binding cassette, sub-family A (ABC1), member 1
Abcg1: ATP binding cassette subfamily G member 1
MMP: Matrix metalloproteinase
DPP-4: dipeptidyl peptidase-4
BODIPY: 4,4-difluoro-4-bora-3a,4a-diaza-s-indacene
vLDL: very low density lipoprotein
Tnf: tumor necrosis factor
Icam1: intercellular adhesion molecule 1

## References

[1] Verma S, Bhatt DL, Bain SC (2018). Effect of liraglutide on cardiovascular events in patients with type 2 diabetes mellitus and polyvascular disease: results of the LEADER trial. Circulation.

[2] Drucker DJ (2018). Mechanisms of action and therapeutic application of glucagon-like peptide-1. Cell Metab.

[3] Pujadas G, Drucker DJ (2016). Vascular biology of glucagon receptor superfamily peptides: mechanistic and clinical relevance. Endocr Rev.

[4] Knudsen LB, Lau J (2019). The discovery and development of liraglutide and semaglutide. Front Endocrinol.

[5] Baggio LL, Yusta B, Mulvihill EE (2018). GLP-1 receptor expression within the human heart. Endocrinology.

[6] Panjwani N, Mulvihill EE, Longuet C (2013). GLP-1 receptor activation indirectly reduces hepatic lipid accumulation but does not attenuate development of atherosclerosis in diabetic male ApoE(-/-) mice. Endocrinology.

[7] Richards P, Parker HE, Adriaenssens AE (2014). Identification and characterization of GLP-1 receptor-expressing cells using a new transgenic mouse model. Diabetes.

[8] Durgin BG, Cherepanova OA, Gomez D (2017). Smooth muscle cell-specific deletion of Col15a1 unexpectedly leads to impaired development of advanced atherosclerotic lesions. Am J Physiol Heart Circ Physiol.

[9] Kim M, Platt MJ, Shibasaki T (2013). GLP-1 receptor activation and Epac2 link atrial natriuretic peptide secretion to control of blood pressure. Nat Med.

[10] Chomczynski P (1992). One-hour downward alkaline capillary transfer for blotting of DNA and RNA. Anal Biochem.

[11] Wong C, Stylianopoulos T, Cui J (2011). Multistage nanoparticle delivery system for deep penetration into tumor tissue. Proc Natl Acad Sci U S A.

[12] Bagalkot V, Badgeley MA, Kampfrath T, Deiuliis JA, Rajagopalan S, Maiseyeu A (2015). Hybrid nanoparticles improve targeting to inflammatory macrophages through phagocytic signals. J Control Release.

[13] Swirski FK, Nahrendorf M, Libby P (2017). Mechanisms of myeloid cell modulation of atherosclerosis. Myeloid Cells in Health and Disease.

[14] Chang W, Zhu F, Zheng H (2019). Glucagon-like peptide-1 receptor agonist dulaglutide prevents ox-LDL-induced adhesion of monocytes to human endothelial cells: an implication in the treatment of atherosclerosis. Mol Immunol.

[15] Pyke C, Heller RS, Kirk RK (2014). GLP-1 receptor localization in monkey and human tissue: novel distribution revealed with extensively validated monoclonal antibody. Endocrinology.

[16] Nakagawa K, Nakashima Y (2018). Pathologic intimal thickening in human atherosclerosis is formed by extracellular accumulation of plasma-derived lipids and dispersion of intimal smooth muscle cells. Atherosclerosis.

[17] Shankman LS, Gomez D, Cherepanova OA (2015). KLF4-dependent phenotypic modulation of smooth muscle cells has a key role in atherosclerotic plaque pathogenesis. Nat Med.

[18] Chen P-Y, Qin L, Li G, Tellides G, Simons M (2016). Smooth muscle FGF/TGFβ cross talk regulates atherosclerosis progression. EMBO Mol Med.

[19] Chen P-Y, Qin L, Li G (2020). Smooth muscle cell reprogramming in aortic aneurysms. Cell Stem Cell.

[20] Vengrenyuk Y, Nishi H, Long X (2015). Cholesterol loading reprograms the microRNA-143/145-myocardin axis to convert aortic smooth muscle cells to a dysfunctional macrophage-like phenotype. Arterioscler Thromb Vasc Biol.

[21] Ninomiya K, Takahashi A, Fujioka Y, Ishikawa Y, Yokoyama M (2006). Transforming growth factor-β signaling enhances transdifferentiation of macrophages into smooth muscle-like cells. Hypertens Res.

[22] Chen P-Y, Qin L, Li G (2019). Endothelial TGF-β signalling drives vascular inflammation and atherosclerosis. Nat Metab.

[23] Wang Y, Dubland JA, Allahverdian S (2019). Smooth muscle cells contribute the majority of foam cells in ApoE (apolipoprotein E)-deficient mouse atherosclerosis. Arterioscler Thromb Vasc Biol.

[24] Bagalkot V, Deiuliis JA, Rajagopalan S, Maiseyeu A (2016). 'Eat me' imaging and therapy. Adv Drug Deliv Rev.

[25] Hoshiga M (2016). Gelatinase and vulnerability of atherosclerotic plaque. J Atheroscler Thromb.

[26] Baggio LL, Drucker DJ (2007). Biology of incretins: GLP-1 and GIP. Gastroenterology.

[27] Qvit N, Rubin SJS, Urban TJ, Mochly-Rosen D, Gross ER (2017). Peptidomimetic therapeutics: scientific approaches and opportunities. Drug Discov Today.

[28] Xiao C, Dash S, Morgantini C, Adeli K, Lewis GF (2015). Gut peptides are novel regulators of intestinal lipoprotein secretion: experimental and pharmacological manipulation of lipoprotein metabolism. Diabetes.

[29] Wu H, Gower RM, Wang H (2009). Functional role of CD11c+ monocytes in atherogenesis associated with hypercholesterolemia. Circulation.

[30] Rakipovski G, Rolin B, Nøhr J (2018). The GLP-1 analogs liraglutide and semaglutide reduce atherosclerosis in ApoE-/- and LDLr-/- mice by a mechanism that includes inflammatory pathways. JACC Basic Transl Sci.

[31] Nandy D, Johnson C, Basu R (2014). The effect of liraglutide on endothelial function in patients with type 2 diabetes. Diab Vasc Dis Res.

[32] Ussher JR, Drucker DJ (2014). Cardiovascular actions of incretin-based therapies. Circ Res.

[33] Siraj MA, Mundil D, Beca S (2020). Cardioprotective GLP-1 metabolite prevents ischemic cardiac injury by inhibiting mitochondrial trifunctional protein-α. J Clin Invest.

[34] Blanco E, Shen H, Ferrari M (2015). Principles of nanoparticle design for overcoming biological barriers to drug delivery. Nat Biotechnol.

[35] Zhang X-Q, Even-Or O, Xu X (2015). Nanoparticles containing a liver X receptor agonist inhibit inflammation and atherosclerosis. Adv Healthc Mater.

[36] Drucker DJ (2016). The cardiovascular biology of glucagon-like peptide-1. Cell Metab.

[37] Arakawa M, Mita T, Azuma K (2010). Inhibition of monocyte adhesion to endothelial cells and attenuation of atherosclerotic lesion by a glucagon-like peptide-1 receptor agonist, exendin-4. Diabetes.

[38] Nagashima M, Watanabe T, Terasaki M (2011). Native incretins prevent the development of atherosclerotic lesions in apolipoprotein E knockout mice. Diabetologia.

[39] Bruen R, Curley S, Kajani S (2017). Liraglutide dictates macrophage phenotype in apolipoprotein E null mice during early atherosclerosis. Cardiovasc Diabetol.

[40] Burgmaier M, Liberman A, Möllmann J (2013). Glucagon-like peptide-1 (GLP-1) and its split products GLP-1(9-37) and GLP-1(28-37) stabilize atherosclerotic lesions in apoe^-^/^-^ mice. Atherosclerosis.

[41] Helmstädter J, Frenis K, Filippou K (2020). Endothelial GLP-1 (glucagon-like peptide-1) receptor mediates cardiovascular protection by liraglutide in mice with experimental arterial hypertension. Arterioscler Thromb Vasc Biol.

