# Supplementary material for: Plaque-targeted, proteolysis-resistant, activatable and MRI-visible nano-GLP-1 receptor agonist targets smooth muscle cell differentiation in atherosclerosis
Source: Theranostics. 2022 Mar 6;12(6):2741–57. doi: 10.7150/thno.66456 (PMC8965488; doi:10.7150/thno.66456)
Supplement: Supplementary file 1 — Supplementary methods, figures and tables. [file thnov12p2741s1.pdf]

Supplementary material online

Plaque-targeted, proteolysis-resistant, activatable and MRI-visible nano-GLP-1 receptor agonist targets smooth muscle cell differentiation in atherosclerosis

Short title: Nano-GLP-1 theranostic for atherosclerosis

Authors: Andrei Maiseyeu[1,2,3,*], Lin Di[1,#], Anastasia Ravodina[1,#], Alma Barajas-Espinosa[2], Atsushi Sakamoto[4], Alice Chaplin[1], Jixin Zhong[1], Huiyun Gao[1], Matthew Mignery[1], Navneet Narula[5], Aloke V. Finn[3,4], Sanjay Rajagopalan[1,2,3]

[1] Case Western Reserve University, Cleveland, OH
[2] Dorothy M. Davis Heart and Lung Research Institute, The Ohio State University, Columbus, OH
[3] University of Maryland, Baltimore, MD
[4] CVPath Institute, Inc., Gaithersburg, MD
[5] NYU Langone Medical Center, New York, NY
[#] These authors contributed equally to this work

***Corresponding author**: Andrei Maiseyeu, PhD, Cardiovascular Research Institute, Case Western Reserve University, School of Medicine, 10900 Euclid Ave, Cleveland, OH 44106
Tel.: 216 368 2005
Fax: 216 368 8898
Email: axm1079@case.edu

Supplementary Figures

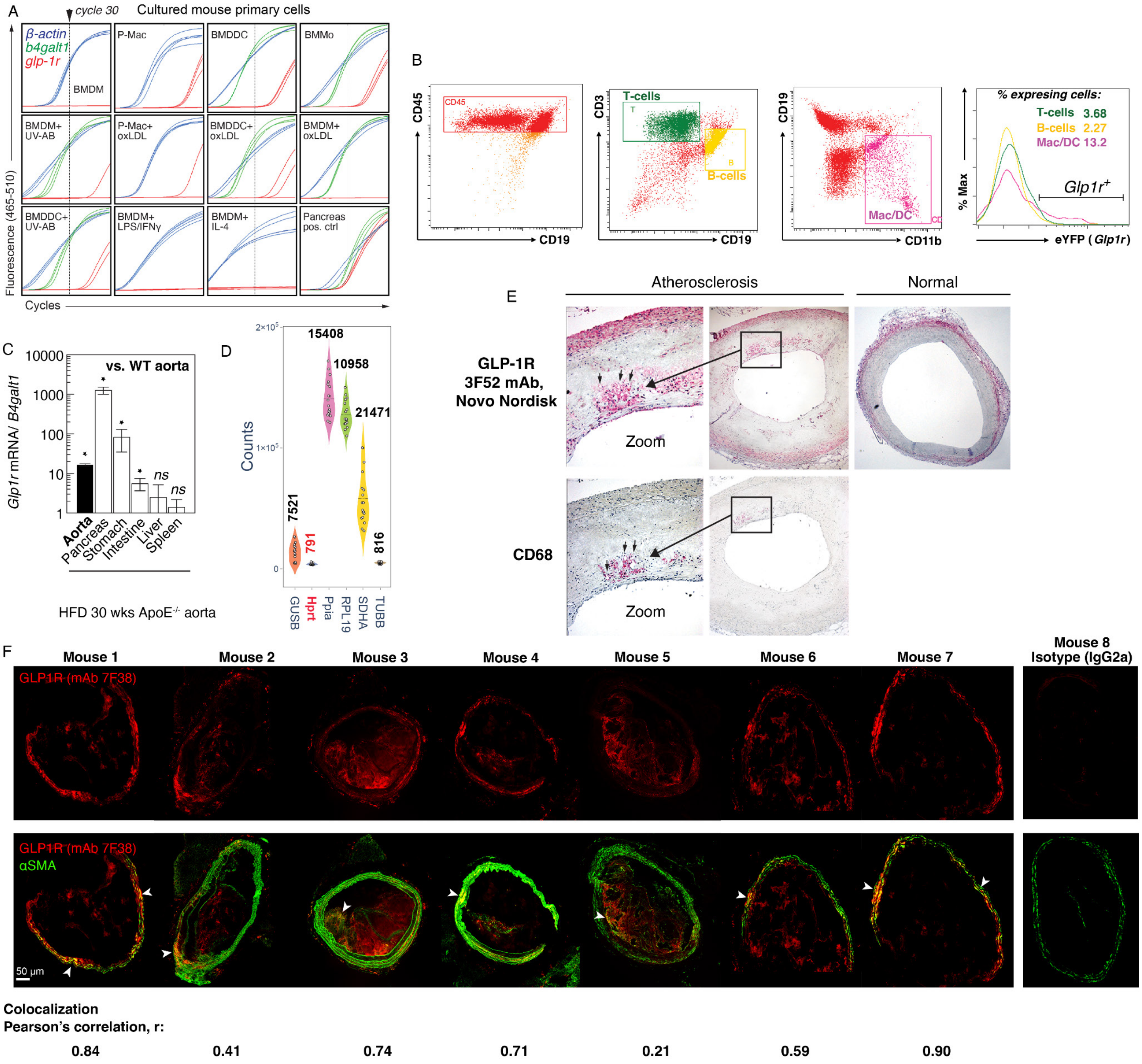

Figure S1. *Glp1r* mRNA expression in mouse and human cells and tissues

A) The expression of Glp1r mRNA transcripts in mouse cultured primary immune cells was null or very low as shown by quantitative real-time PCR amplification curves. The cutoff was set at the 30th cycle (arrowhead and dotted line) for reliable analysis. Atherosclerosis-relevant conditions were tested: BMDM, P-Mac, BMDDC, BMMo - bone marrow derived macrophages, peritoneal macrophages, bone marrow derived dendritic cells and monocytes respectively, were cultured at high glucose conditions or additionally treated with 0.5 μg/mL oxidized LDL (oxLDL), 10^4 /10^6 cells UV-induced apoptotic bodies (UV-AB), 100 ng/mL LPS plus 100 U/mL interferon gamma (LPS/IFNγ), or 10 ng/mL interleukin 4 (IL-4). Whole pancreas mRNA served as a *Glp1r* positive control. **B)** Splenocytes from mice expressing Cre recombinase under the Glp1r promoter crossed with a ROSA26 eYFP (Glp1r-Cre/R26R-EYFP) were analyzed by flow cytometry co-staining with antibodies against CD45 (pan myeloid cells), CD19 (B cells), CD3 (T cells) and CD11b (macrophages). Gating was selected as indicated. Glp1r$^+$ cells were detected in gated populations through intrinsic eYFP signal. **C)** Glp1r mRNA expression in mouse whole atherosclerotic aortas is comparable to those in organs known to express Glp1r. Relative mRNA expression is calculated vs. control ΔCt values obtained from the whole aorta of C57BL/6 mouse. **D)** The expression of various housekeeping genes among 30 atherosclerotic tissues was analyzed by

Nanostring (https://www.nanostring.com). Corresponding counts are shown as averages (horizontal dotted line), violin shape is representative of data distribution and standard deviations are depicted in bold numbers. Hypoxanthine-guanine phosphoribosyltransferase (Hprt, highlighted in red) was chosen for Southern blot normalization. **E)** GLP-1R staining (anti-GLP-1R Mab 3F52, Novo Nordisk) was observed in human coronary artery tissue obtained from autopsy cases in normal controls and atherosclerosis and co-stained with anti-CD68 (red staining). **F**) Immunofluorescence staining of brachiocephalic artery (BCA) sections from seven Apoe$^{-/-}$ mice high-fat fed for 30 weeks using anti-mouse monoclonal GLP-1R antibody mAb 7F38 (red). The sections were co-stained with goat anti-α-smooth muscle actin (αSMA), which is displayed in green. Pearson's correlation coefficient of colocalization numbers are shown at the bottom. White arrowheads point to sites of most prominent GLP-1R expression.

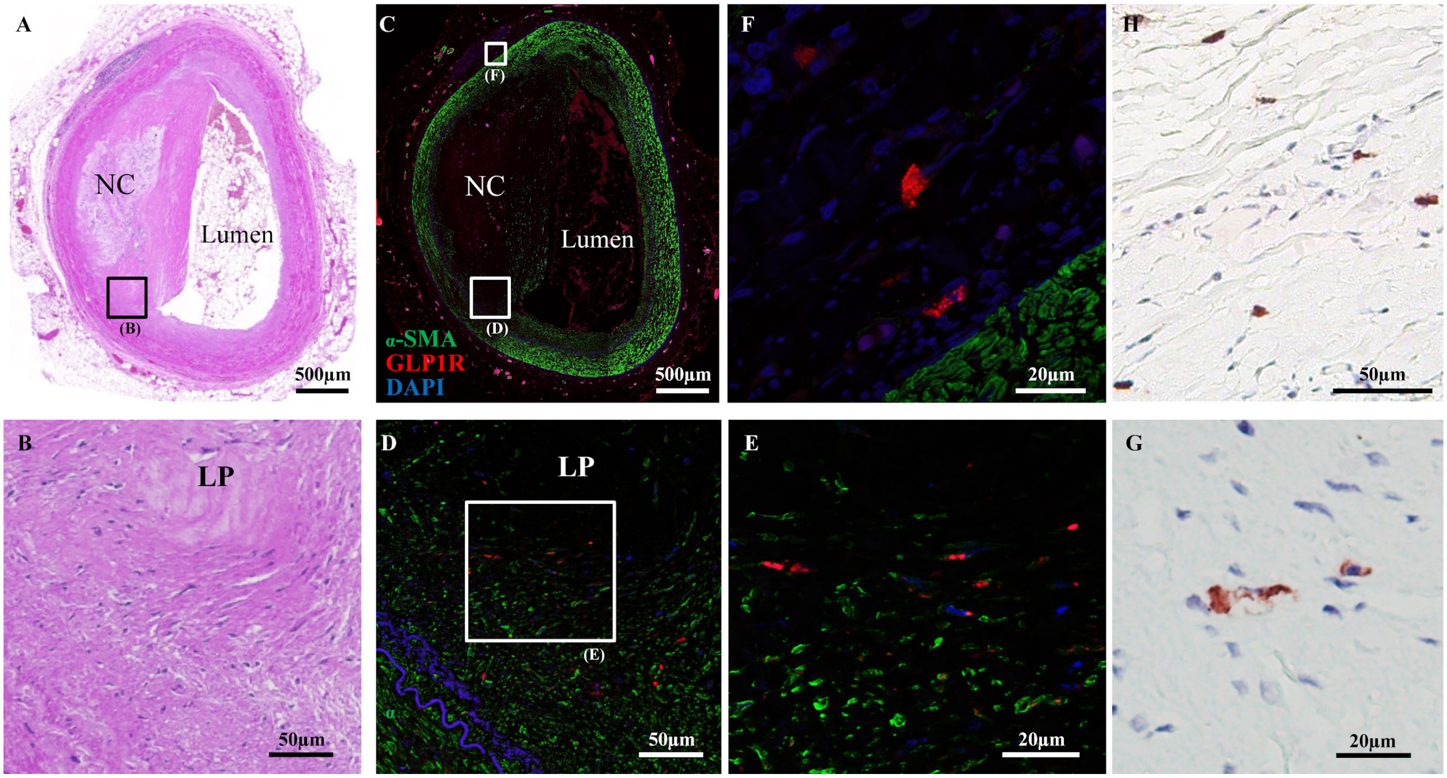

Figure S2. Human coronary artery pathology obtained from 49 years old white male autopsy case who has severe three vessel coronary atherosclerotic disease. A) Low power H&E staining section of atherosclerotic coronary artery (early fibroatheroma) in proximal LAD. **B)** Middle power H&E image of black rectangular field in A. The field focused on the surrounding area of LP. **C)** IF staining image of αSMA (green) and GLP1R (red) with DAPI nuclear staining (blue) which is adjacent section of A. **(D-E)** Middle power (D) and high power (E) image of white rectangular field in C and D. Spindle shape with GLP1R (red) positive cells were observed in the field. **F)** High power image of adventitial lesion at white rectangular field in C. Multiple GLP1R positive cells were observed. **(G-H)** IHC image of GLP1R positive cell (brown) in the field of intra-plaque (G) and adventitia (H) which is obtained from adjacent fields of E and F. *Abbreviations:* H&E - hematoxylin and eosin; LAD - left anterior descending artery; NC - necrotic core; LP - lipid pool; αSMA - alpha smooth muscle actin; GLP1R - glucagon-like peptide receptor 1; DAPI - 4′,6-diamidino-2-phenylindole.

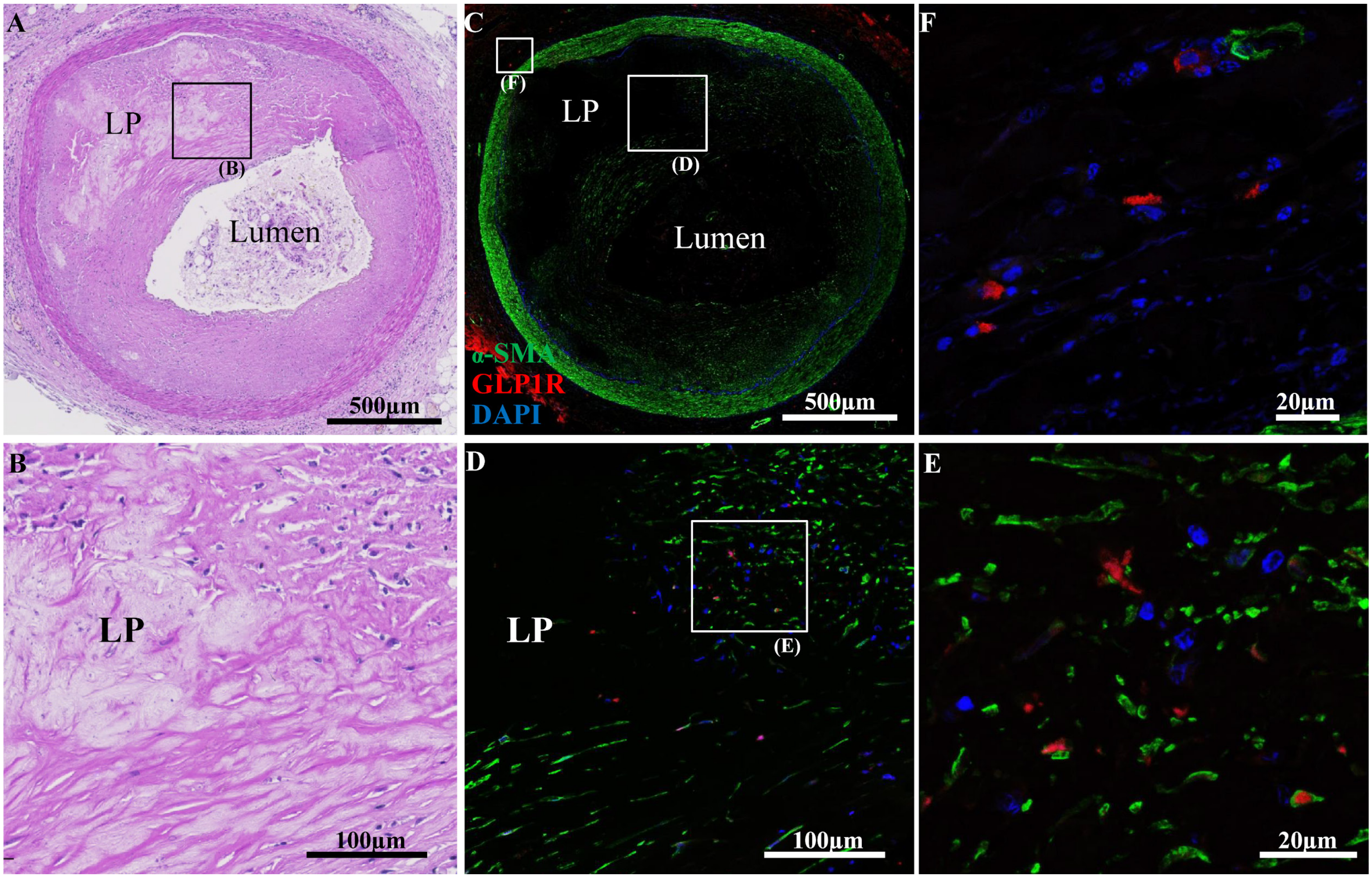

Figure S3. Human coronary artery pathology obtained from a 57 years old white female who suddenly died with acute myocardial infarction. A) Low power H&E staining section of atherosclerotic coronary artery (pathologic intimal thickening) in mid LCX. **B)** Middle power H&E image of black rectangular field in A. **C)** Immunofluorescent (IF) staining image of αSMA (green) and GLP1R (red), counterstaining nuclei with DAPI (blue). (**D-E**): Middle power (D) and high power (E) images of white rectangular field in C and D. GLP1R positive cells (red) were observed beside the LP area. **F)** High power image of adventitial lesion at white rectangular field in C. *Abbreviations:* H&E - hematoxylin and eosin; LAD - left anterior descending artery; LP - lipid pool; LCX - left circumflex artery; αSMA - alpha smooth muscle actin; GLP1R - glucagon-like peptide receptor 1; DAPI - 4′,6-diamidino-2-phenylindole.

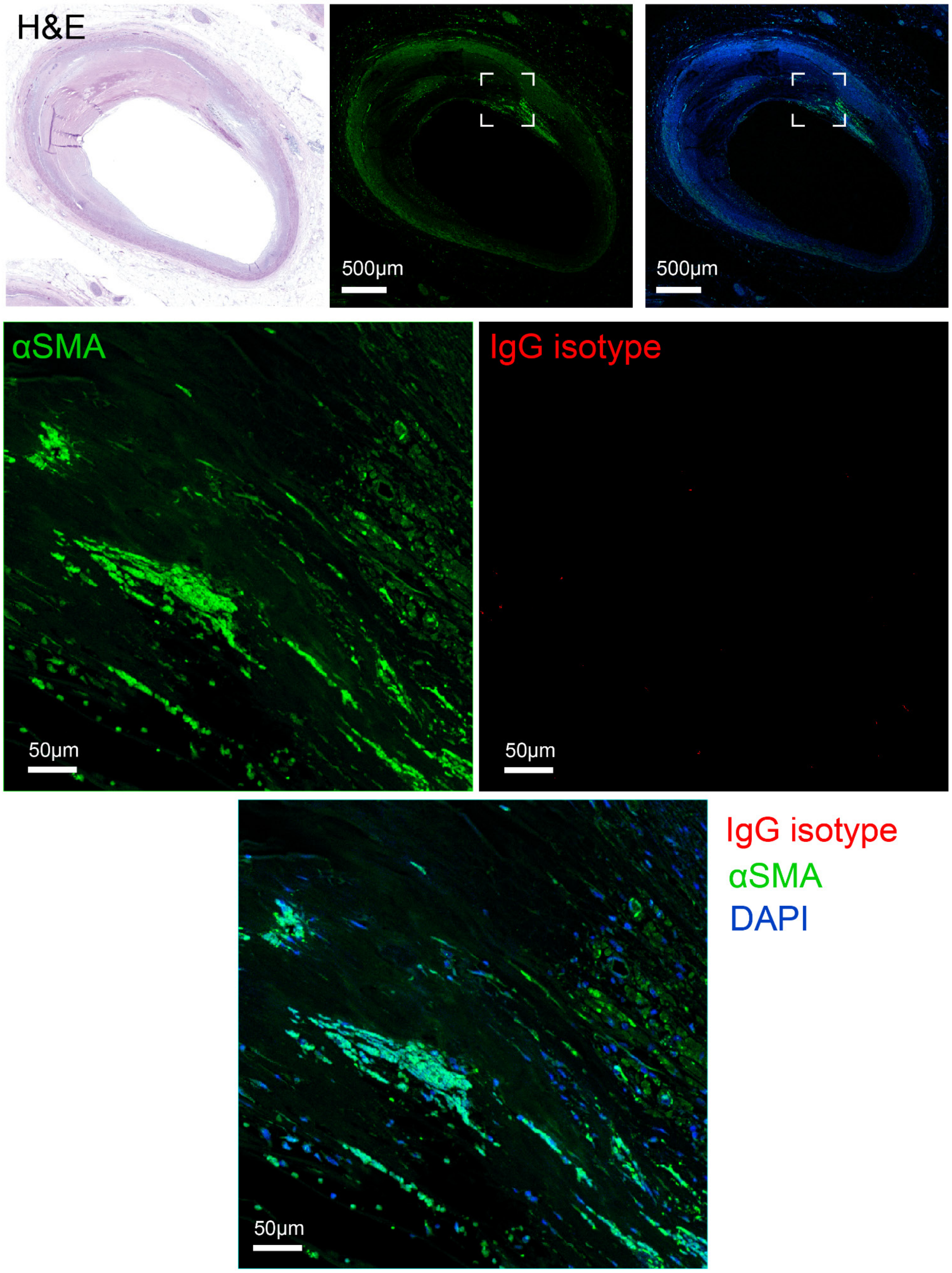

Figure S4. Isotype control staining in human coronary artery. Immunofluorescent staining images of αSMA (green) and mouse IgG1 (red, isotype for GLP1R) are shown. Nuclei were counterstained with DAPI (blue).

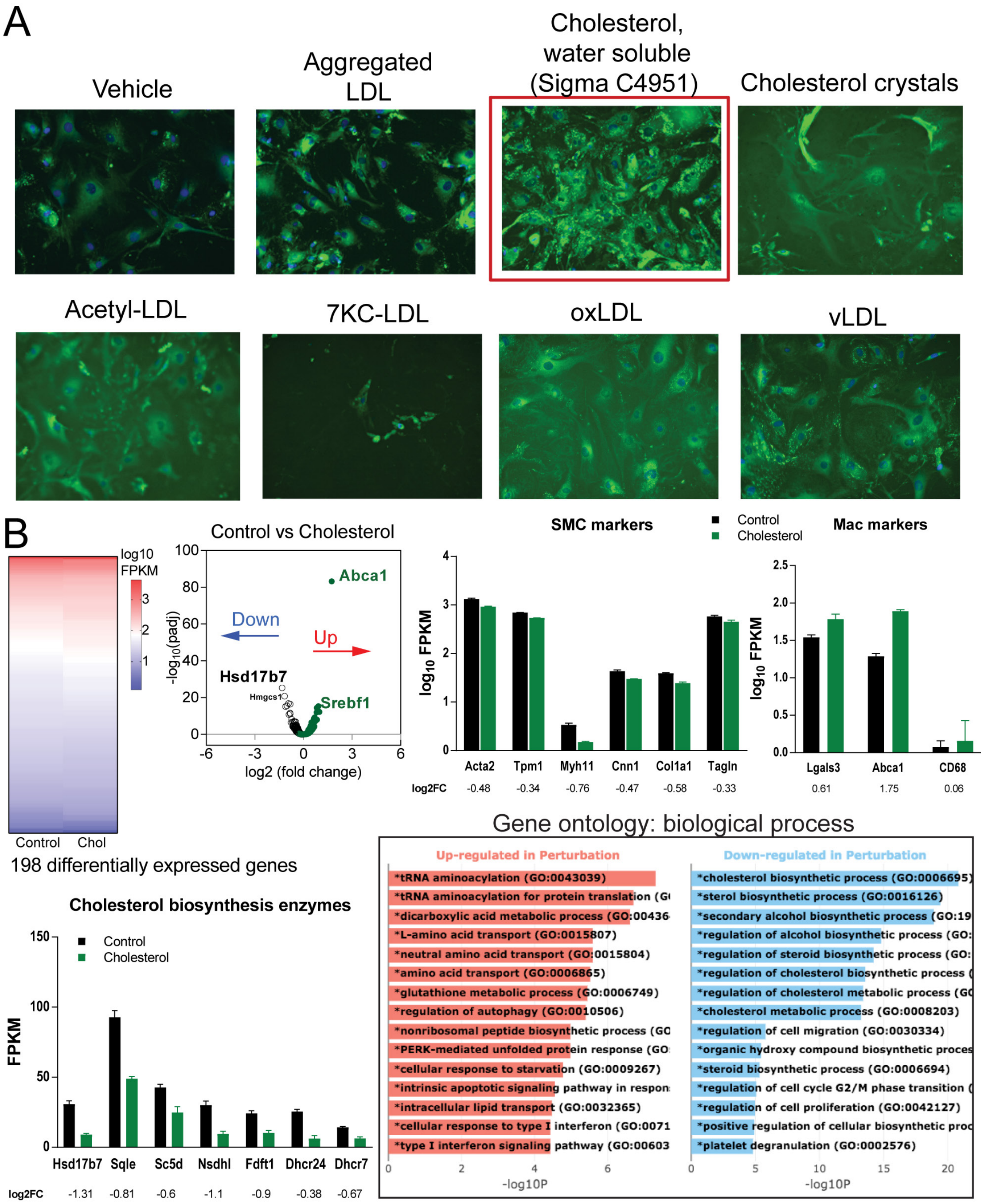

Figure S5. Specific cholesterol loading conditions induce mouse aortic smooth muscle cells (MASMC) de-differentiation. A) Various cholesterol loading conditions were tested to overload MASMC with cholesterol as indicated. Modified low density lipoproteins (LDL), including aggregated, acetylated (Acetyl-), loaded with 7-ketocholesterol (7KC), oxidized (oxLDL) and very low density (vLDL) were incubated with MASMC at 100 µg/mL for 96 h. This was compared with MASMC incubated with free soluble cholesterol (cyclodextrin complex, Sigma C4951) at 10 µg/mL or cholesterol crystals at 200 µg/mL. Intracellular cholesterol deposition was analyzed by BODIPY staining (green) after cellular fixation. Nuclei were counterstained with Hoescht (blue). **B)** The MASMC phenotype was confirmed by RNA sequencing (RNAseq) after loading with C4951 for 96 h. 198 differentially expressed genes were found, including downregulated SMC markers and upregulated macrophage (Mac) markers as indicated in bar graphs. Major cholesterol biosynthesis genes were downregulated. Gene ontology pathway analysis indicated various cholesterol biosynthesis pathways significantly downregulated, however lipid transport, autophagy and inflammation upregulated. Averages from two independent experiments (two untreated and two C4951-treated) are shown with an error bar indicating the spread of the data.

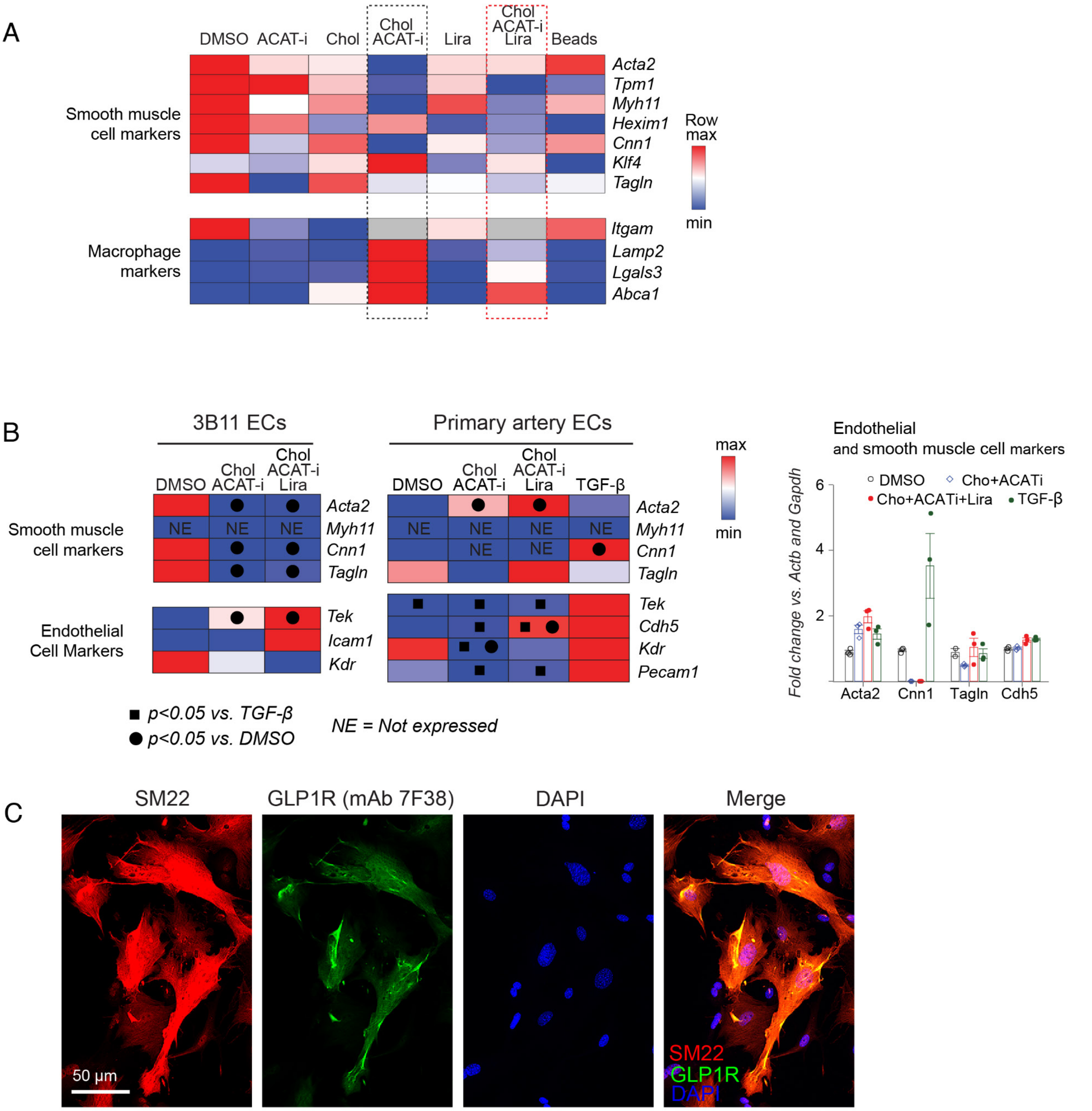

Figure S6. Phenotypic changes in smooth muscle and endothelial cells under cholesterol loading conditions *in vitro*. A) Heatmap representing the relative expression of indicated genes in primary smooth muscle cells treated as indicated and analyzed by quantitative PCR. The absolute values of expression change and corresponding statistical analysis can be found in Supplementary Table S4. **B)** Heatmap representing the expression of smooth muscle and endothelial cell markers in the endothelial cell line 3B11 and primary carotid artery endothelial cells treated as indicated and analyzed by quantitative PCR (left). Statistically significant differences between groups are indicated by squares and circles and additionally plotted as a bar graph (right). NE - not expressed. **C)** Immunofluorescence staining in primary mouse aortic smooth muscle cells (MASMCs) using antibodies against a marker of adult smooth muscle SM22 (transgelin, red) and co-staining with anti-mouse GLP-1R mAb 7F38 (green). Nuclei were counterstained with DAPI (blue).

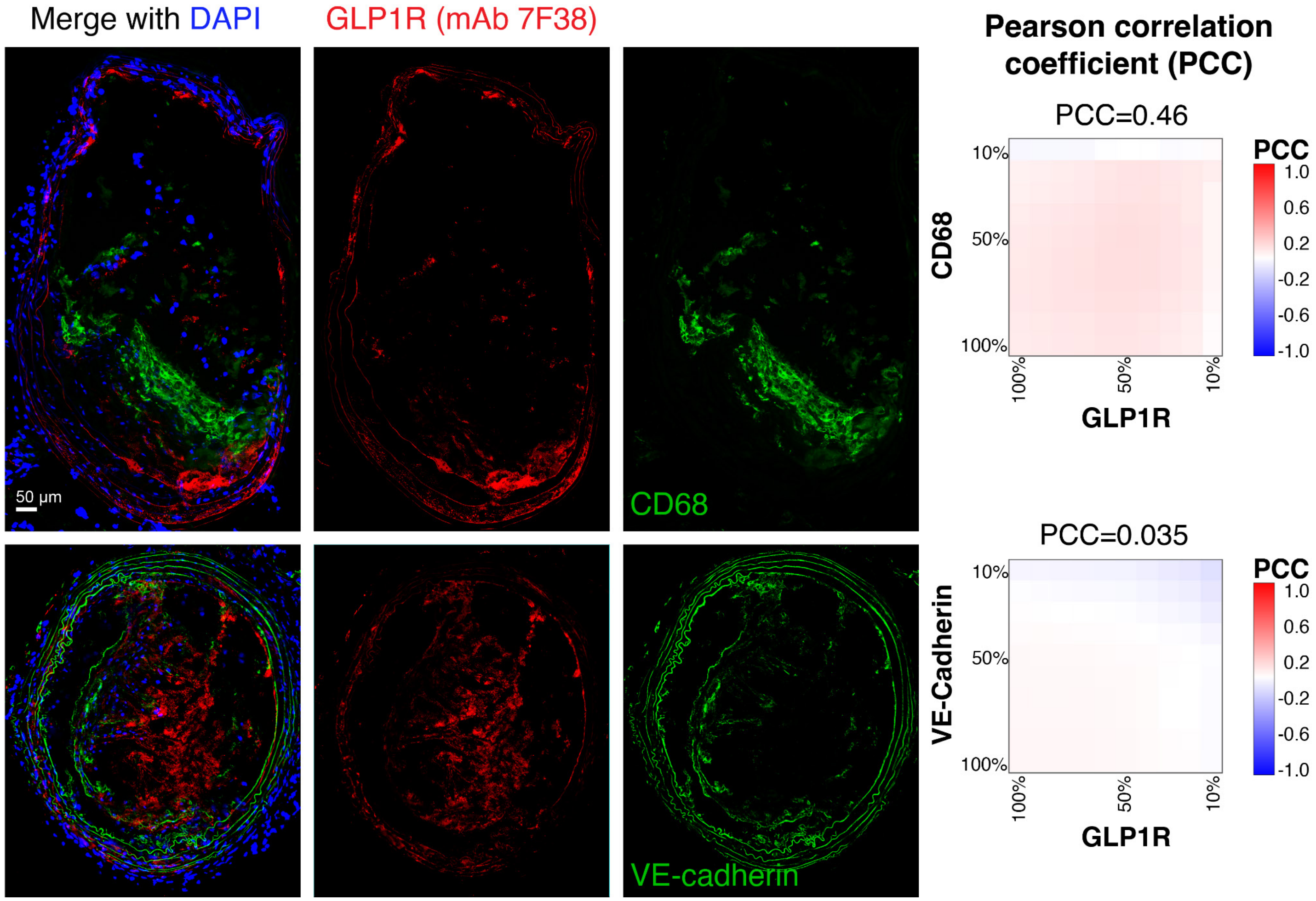

Figure S7. Immunofluorescence analysis of GLP-1R expression in mouse atherosclerosis. Immunofluorescence staining was performed on brachiocephalic artery (BCA) sections from Apoe$^{-/-}$ mice high-fat fed for 30 weeks using anti-mouse monoclonal GLP-1R antibody mAb 7F38 (red). The sections were co-stained with rabbit anti-CD68 (macrophage marker, green) displayed at the top set of images, or with rat anti-VE-cadherin (endothelial cell marker, green) at the bottom set of images, as indicated. Corresponding Pearson's correlation coefficients of colocalization are shown at the right. Metric matrices for two reporter channels were calculated using Pearson correlation coefficient (PCC) and show percentile (%) of pixels for signal intensity in a given image. PCC=1 means complete co-localization and PCC=-1 defines complete lack of co-localization.

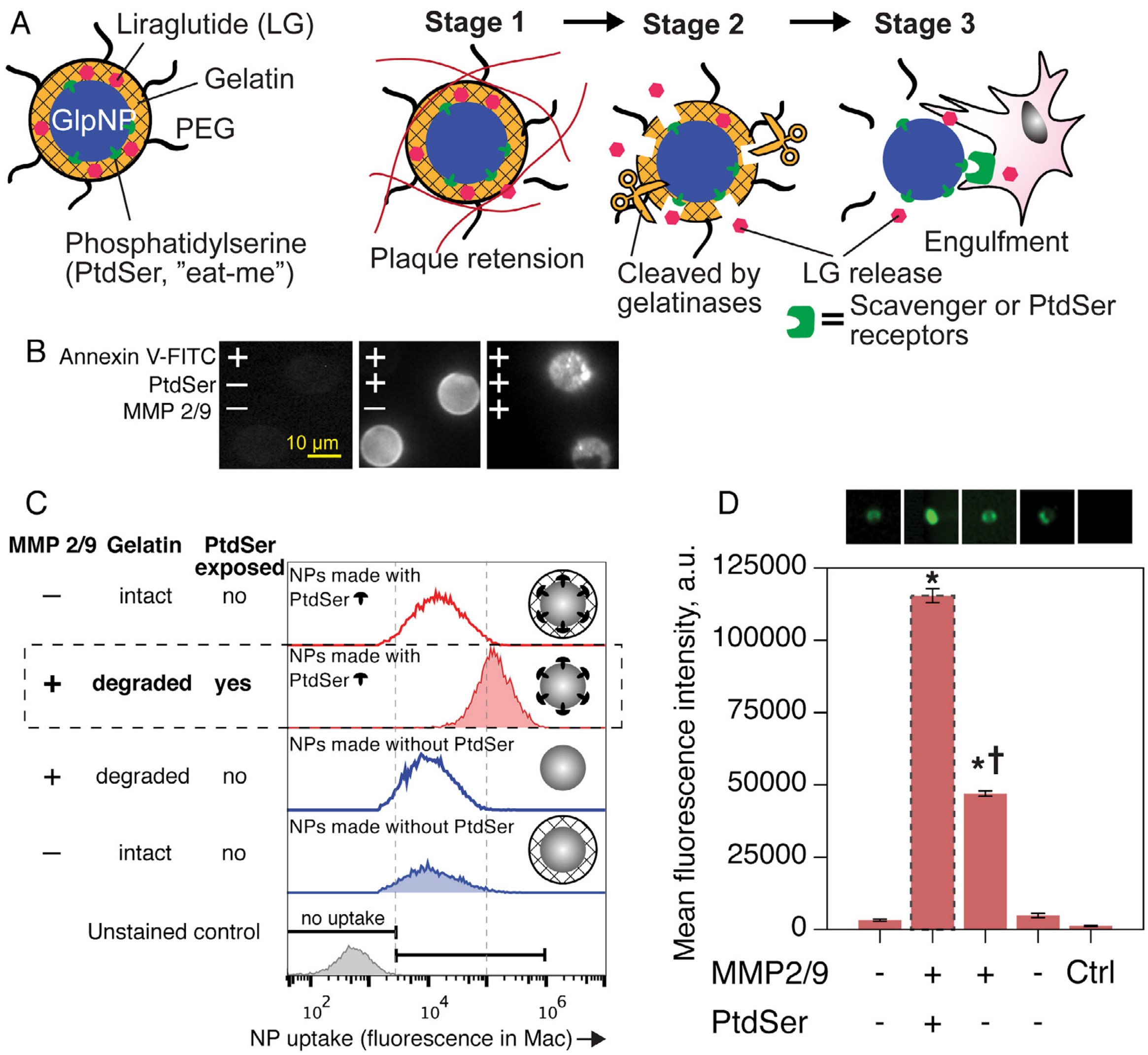

Figure S8. A) Proposed mechanism of action of activatable GlpNP. GlpNP has three layers: i) latex (polystyrene) core with fluorochrome (blue), ii) gelatin (orange), iii) polyethylene glycol (black), iv) phosphatidylserine (green) and liraglutide (red). The structure (as depicted) and mechanism of activation of GlpNP are hypothetical and speculated based on GlpNP physico-chemical characterization (see Supplementary Methods), the data below and the data presented in the main text. **B)** In a cell-free model system, biotin-containing non-fluorescent GlpNP were immobilized on 10 µm avidin latex beads, followed by annexin V-FITC staining in the presence or absence of gelatinases (cocktail of MMP-2 and MMP-9). GlpNP without gelatin coating showed uniform annexin V staining while gelatin coated GlpNP decorated with PtdSer demonstrated punctate annexin V staining following MMP-2/MMP-9 incubation, indicating degradation of the gelatin and binding of annexin V to the exposed PtdSer Fluorescence micrographs showed prominent punctate Annexin V-FITC staining of GlpNP decorated with phosphatidylserine (PtdSer+) and gelatinase-degraded gelatin (Gelatin + MMP) [far right]. Nanoparticles without gelatin coating but with PtdSer+ showed uniform staining [middle]. No staining was observed in the absence of PtdSer [left]. **C)** GlpNP that contained both PtdSer and degraded gelatin coating were engulfed rapidly. Low uptake was noted with an intact gelatin layer (PtdSer is not exposed) in the absence of MMP-2/MMP-9 **D)** Macrophages engulfed GlpNP that contained both PtdSer and degraded gelatin coating, but not GlpNP where gelatin was intact. Uptake efficiency was quantified by imaging flow-cytometry after treatment of bone marrow-derived macrophages with nanoparticles in the presence or absence of MMP2/MMP9.

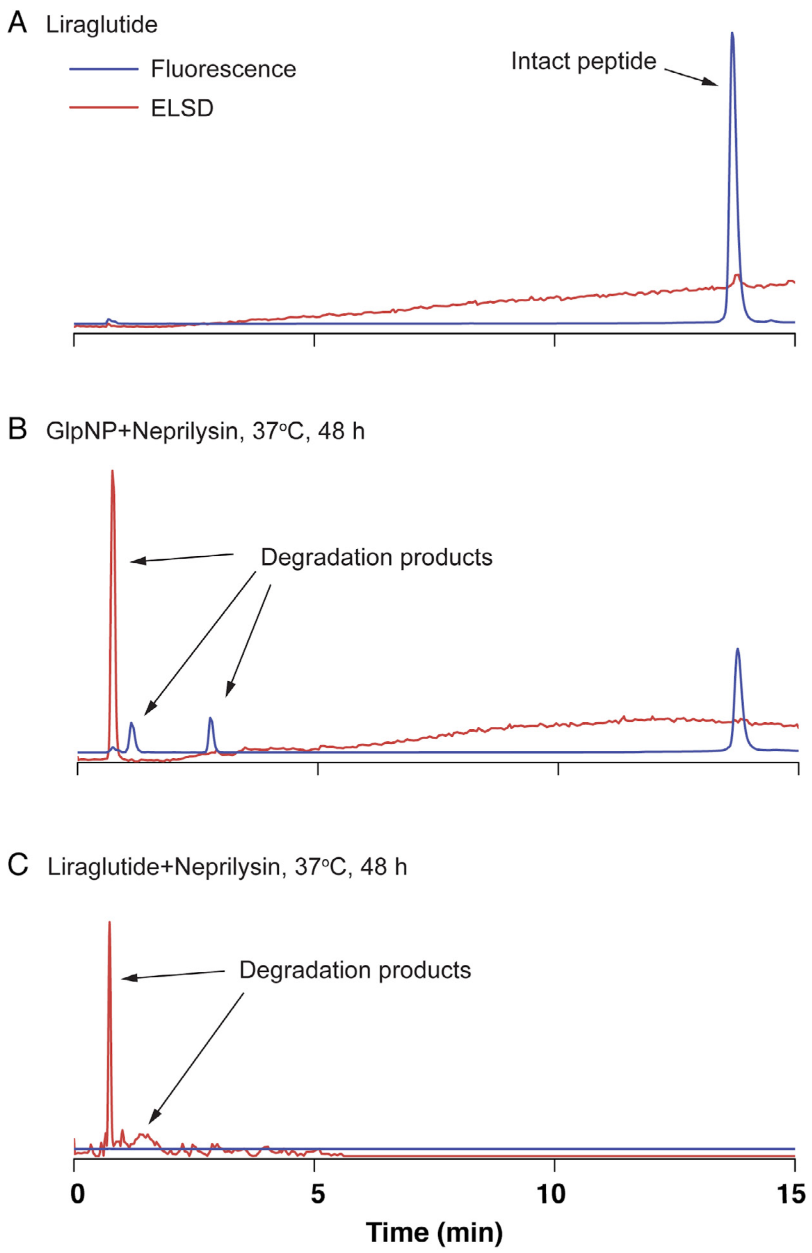

Figure S9. Representative chromatograms from analysis of GlpNP degradation in the presence of neutral endopeptidase (NEP, Neprilysin). A) Before enzyme addition**, B)** GlpNP 48 h after Neprilysin was added, **C)** Liraglutide 48 h after Neprilysin was added. The analysis was performed as described in Methods using two detection modes simultaneously registering liraglutide fluorescence with a fluorescence detector (280 nm excitation and 350 nm emission) and with an evaporative light scattering detector (ELSD). Intact peptide is highly fluorescent, however the products of its degradation (small peptide fragments, amino acids) maybe not and are detected with ELSD.

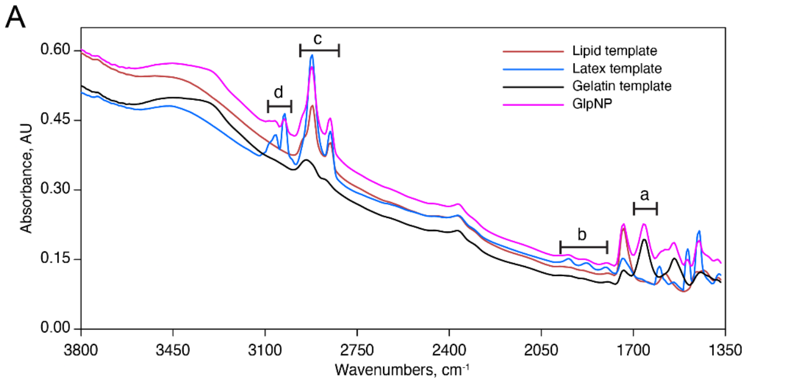

Label	Group	Absorption (cm^{-1})	Identity
a	Amide bond	1600−1690	Protein
b	Benzene fingers	1750−2000	Polystyrene
c	Symmetric/asymmetric CH2 stretch	2850−2925	Polystyrene, lipid
d	Aromatic C-H stretch	3081, 3059, 3025	Polystyrene

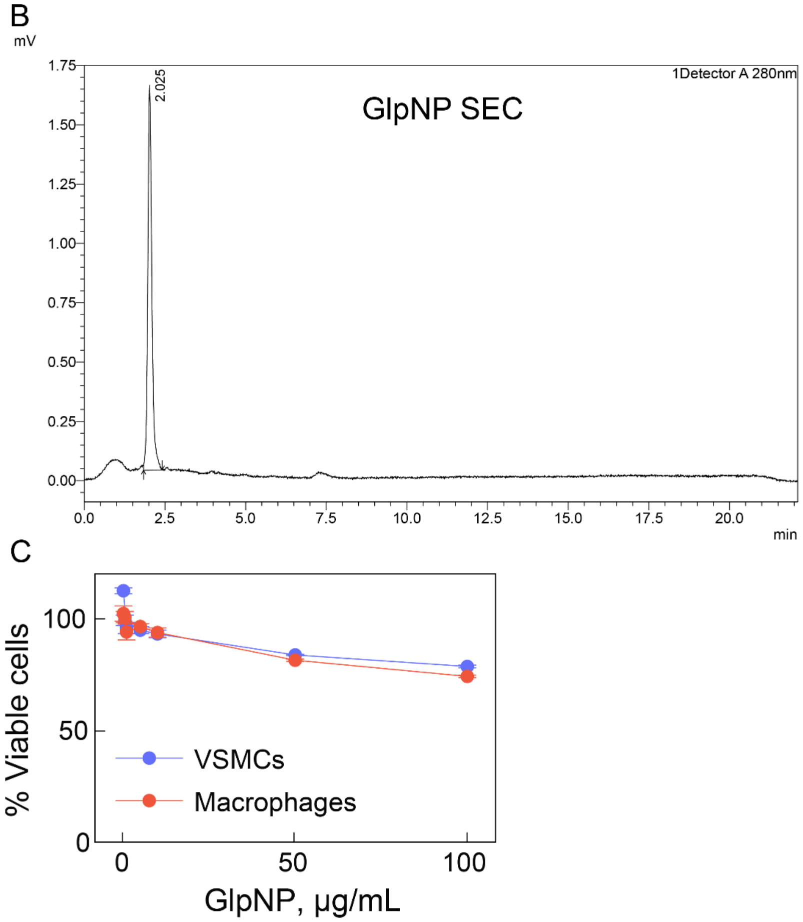

Figure S10. Physicochemical characterization of GlpNP. A) Fourier transformed infrared spectra (FTIR) of GlpNP and its individual constituents: PtdSer lipids, polystyrene latex, and gelatin. Characteristic IR absorption wavenumbers and their identity are assigned with labels a-d are shown in the table. GlpNP exhibited characteristic IR signature present in lipid, polystyrene and gelatin. **B**) Size exclusion chromatography (SEC) of GlpNP. The 50 µL injection of 0.5 mg/mL GlpNP in Tosoh SEC TSKgel G5000PXL column detected a single peak at 2.025 min, demonstrating the integrity of GlpNP particles. **C**) Lactate dehydrogenase (LDH) release assays were performed in primary vascular smooth muscle cells and bone marrow derived macrophages over the range of GlpNP concentrations. GlpNP was not toxic achieving >90% cell viability with the dose up to 50 µg/mL.

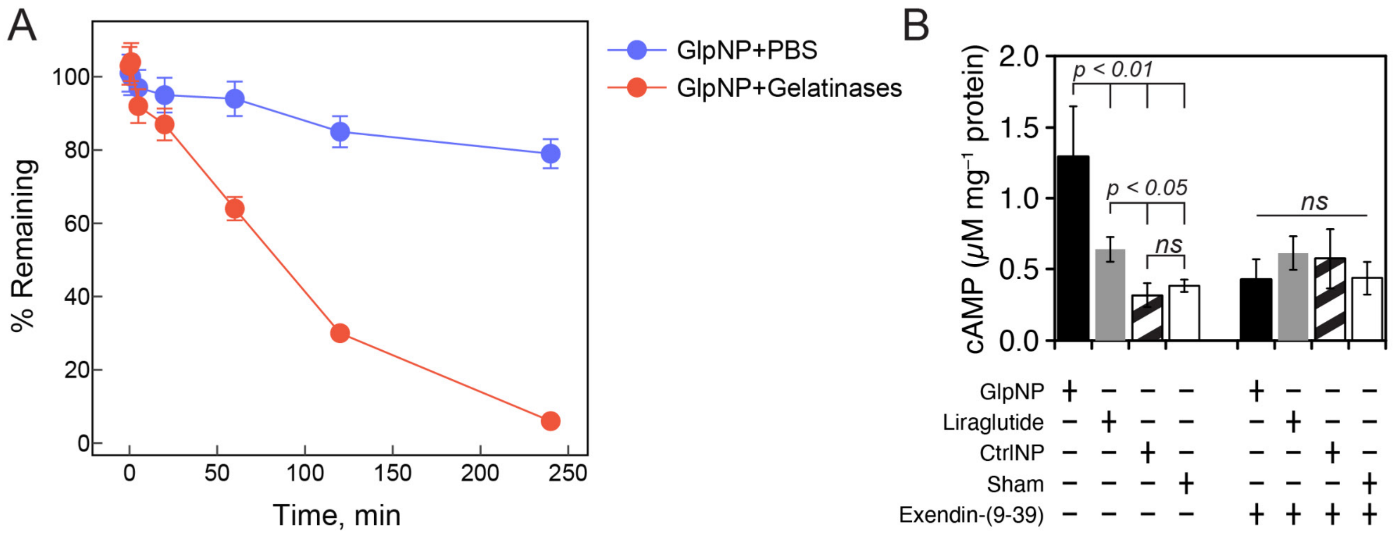

Figure S11. GlpNP show bioactivity *in vitro*. A) Cell-free liraglutide release studies with GlpNP treated with PBS or a cocktail of gelatinases MMP2 and MMP9. After incubation at 37°C for an indicated time period, the GlpNP were pelleted by ultracentrifugation at 100,000 g (10 min, 4°C) and extracted with acetonitrile/water. The extracts were then analyzed on liraglutide concentration using HPLC as described in Methods. The data are presented as a percent of liraglutide remaining in the original GlpNP particles extracted at time zero. **B**) GlpNP (at 100 pM based on incorporated Lira) incubated with RINm5F rat pancreatic islet insulinoma cells showed potent cAMP-inducing activity, a prototypical response to GLP-1R-mediated signaling. This effect was blunted in the presence of the excess (100 nM) exendin-(9-39), an antagonist of GLP-1R signaling. cAMP levels were analyzed in the presence and absence of excess (100 nM) exendin (9–39), an antagonist to GLP-1R. Liraglutide and GlpNP were used at 100 pM (based on liraglutide concentration in GlpNP). *p<0.05 vs liraglutide.

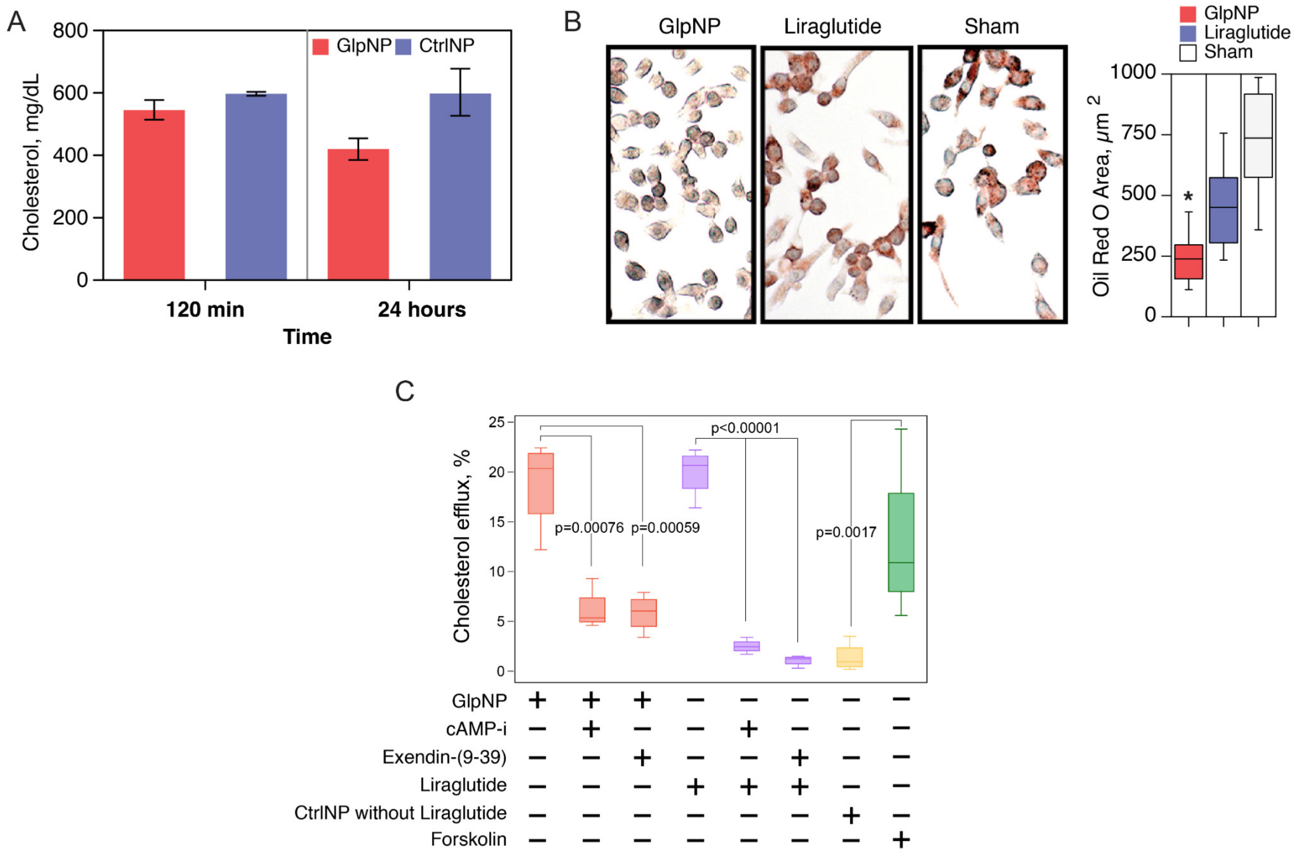

Figure S12. A) Total cholesterol content in plasma after 120 min and 24 h post GlpNP or CtrlNP administration. **B)** Lipid accumulation in human primary aortic smooth muscle cells (HASMC), pre-treated with GlpNP, liraglutide or PBS control followed by acLDL loading for 48 h and oil red o staining. **C)** Cholesterol efflux studies performed in HASMC. The cells were pretreated as indicated and the experiments were performed as described in Supplemental Methods. n=4-6, *P<0.05 vs. GlpNP alone; †P<0.05 vs. liraglutide alone.

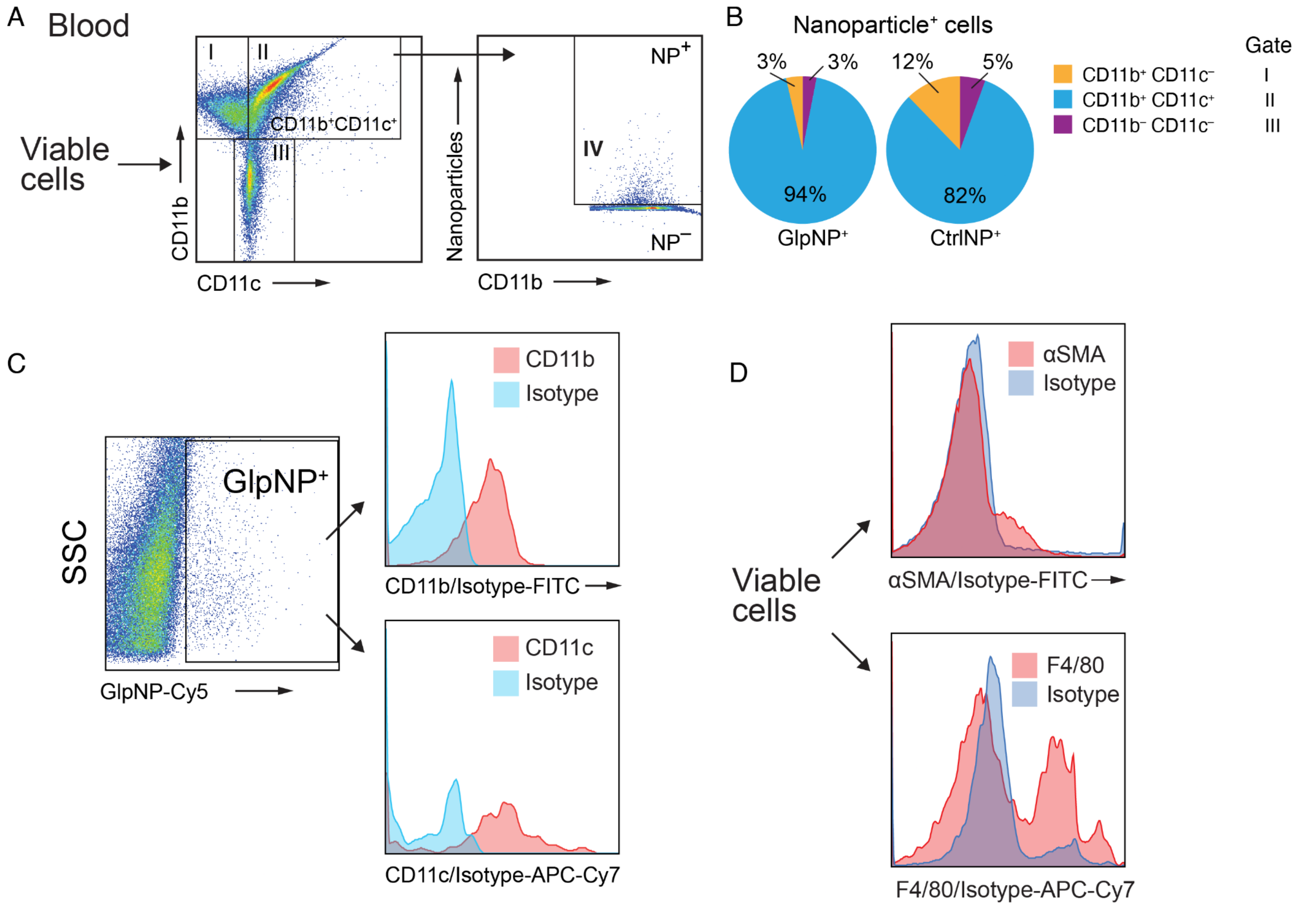

Figure S13. GlpNP target myeloid and SMCs in atherosclerosis. A, B) GlpNP and CtrlNP preferentially target CD11b⁺CD11c⁺ immune cells in blood as assessed by flow cytometry. Both nanoparticle (NP) types are equally retained within this cell population in blood 24 h after bolus intravenous injection in high-fat fed Apoe-/- mice as described in the text and methods. **B**) The pie diagram demonstrates total NP positivity among indicated cell types. **C, D**) Isotype controls for CD11b, CD11c, F4/80 and αSMA antibodies used for flow-cytometric assessment of GlpNP targeting of aortic cells. Main histograms with indicated markers are shown in Figure S13A and the main text Figure 4A,B.

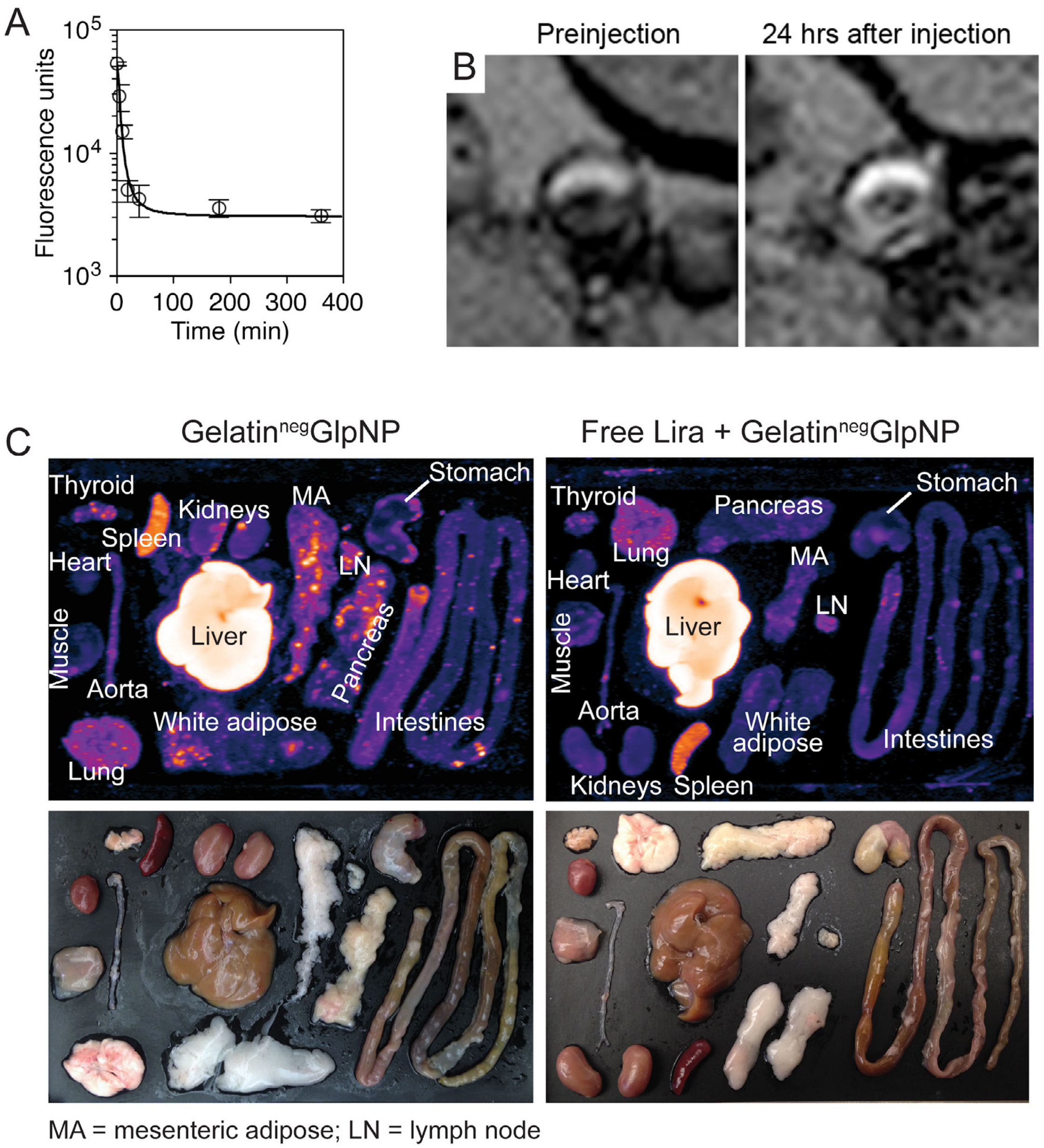

Figure S14. A) Pharmacokinetic behaviour of GlpNP after injection in Apoe$^{-/-}$ mice and analysis using fluorescence intensity of blood samples. **B)** Accumulation of CtrlNP (liraglutide^{-}, PtSer^{+}) in atherosclerosis as evidenced by MRI 24 h after injection. **C)** Accumulation of GlpNP without gelatin coating (gelatin negative [Gelatin^{neg}] GlpNP) in various organs as assessed by fluorescence imaging of whole extracted organs. Gelatin^{neg}GlpNP were administered at 30 µg/kg (based on liraglutide) via bolus i.v. injection (left panel) and the mice were euthanized 24 h later. Gravity perfusion for at least 10 min with citrate-PBS was used to clear any non-engulfed particles before organ collection. In a separate experiment, 300 µg/kg of free liraglutide was administered simultaneously with 30 µg/kg Gelatin^{neg}GlpNP (right panel) and the organs were imaged as above.

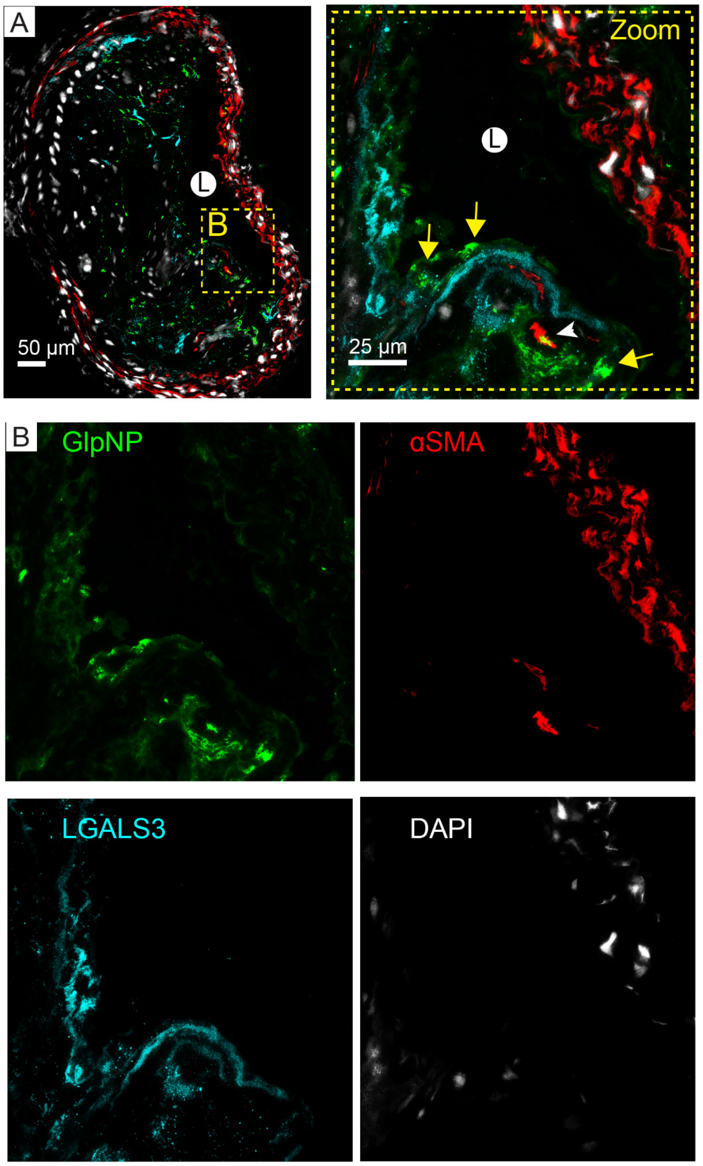

Figure S15. Immunofluorescence analysis of nanoparticle cellular distribution in atherosclerosis. A,B) GlpNP localization in BCA 24 h after bolus injection in high-fat fed Apoe$^{-/-}$ mice as described in the text and methods. Frozen aortic root sections were stained with antibodies against LGALS3 (Mac 2, Galectin-3, cyan) and αSMA (red) and co-visualized with endogenous Cy5 signal from GlpNP (green). The pattern of GlpNP distribution (arrows) was within αSMA+ fibrous cap, which was also enriched in macrophage-like LGALS3+ cells. Nuclei (blue) were stained with DAPI.

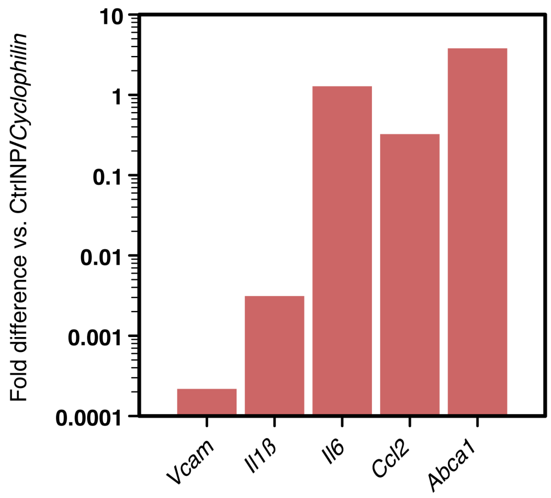

Figure S16. Gene expression analysis of indicated targets was quantitatively assessed after laser capture microdissection (LCM). The values presented are the average of two aortic samples. RNA was collected from 30-40 LCM slides.

Supplementary Methods

Materials, supplies and general methods

Extensively validated monoclonal anti-human GLP1R antibody (MAb 3F52)[1] was a kind gift from Novo Nordisk, Denmark. Lipoproteins LDL, oxLDL, and HDL were from Athens Research and Technology (Athens, GA). Liraglutide was from Bachem Americas (Torrance, CA). PCR reagents and cell culture supplies were from Life Technologies as indicated (see Supplementary Table S1). Mouse antibodies (Supplementary Table S1) were from Abcam (Cambridge, MA), Thermo Fisher, and BD Biosciences. Bone marrow and peritoneal macrophages (BMDMs and P-Mac respectively) were obtained according to published protocols and as previously described by us [2–4]. HPLC solvents were LCMS Optima grade and were from Thermo Fisher. Insulin concentration was analyzed using ultra-sensitive ELISA (Crystalchem, Elk Grove Village, IL). Cyclic AMP was measured by an ELISA kit from Cayman Chemical (Ann Arbor, MI). Mouse Apolipoprotein B48 ELISA Kit was from MyBioSource, Inc. (San Diego, CA). Laser capture microdissection was performed by The Laser Capture Molecular (LCM) Core of The Ohio State University Medical Center (OSUMC). Myograph experiments were performed on thoracic aortas as previously described by us.[5] Tissue processing, staining and immunohistochemistry was performed by the Case Western Reserve University Institute of Pathology core laboratory. Magnetic resonance imaging of the abdominal aorta was performed as previously described by us [4,6] and others [7–9] using Bruker BioSpec 9.4T horizontal bore scanner.

Immunohistochemistry of human plaque

Human tissues were obtained from autopsy specimens, under exemptions granted by the Institutional Review Boards of the New York-Presbyterian Weill Cornell Medical Center. Immunohistochemical staining was performed using GLP1R (MAb 3F52, Novo Nordisk, Denmark) staining in human coronaries that were formalin-fixed and paraffin-embedded. Staining was performed on the 5 micron tissue sections on a Leica Bond system using the standard protocol and as described by us.[10] The sections were pre-treated using heat mediated antigen retrieval with a Tris-EDTA buffer at pH 9 for 20 min. The sections were then incubated with MAb 3F52 (1:1000 dilution) for 15 min at room temperature and detected using an HRP conjugated compact polymer system. 3, 3-Diaminobenzidine (DAB) was used as the chromogen. The sections were then counterstained with haematoxylin and mounted with Leica Micromount. Alternatively, immunohistochemistry was performed on the Ventana Discovery system (Roche). Primary anti-human GLP1R mouse antibody (Mab 3F52, Developmental Studies Hybridoma Bank) was used at 1:5 dilution. The staining was developed and visualized by using the NovaRED kit (Vector Laboratories).

Immunofluorescence in human plaque

Formalin fixed paraffin-embedded coronary artery tissues were selected from the CVPath autopsy registry. Histologic sections were cut on a rotary microtome at 5 to 6 μm, mounted on charged slides, and stained with hematoxylin and eosin (H&E) for tissue characterization.

Primary anti-human αSMA rabbit antibody obtained from Abcam (ab5694) was used with 1:1000 dilution. Primary anti-human GLP1R mouse antibody (Mab 3F52, Developmental Studies Hybridoma Bank) was used in 1:5 dilutions. Alexa Fluor-labeled secondary antibodies (Life Technologies) were used for further incubation. Tissue was visualized using an objective on a LSM 800 laser scanning confocal microscopy (Zeiss). Projection images were generated by collecting the maximum pixel intensity from each image of the Z stack and by projecting pixel intensity onto the single (projection) image.

Human peripheral blood monocytes (PBMC) isolation and differentiation

PBMC isolation and culture was performed as previously described.[11,12] Briefly, blood was drawn from healthy volunteers into BD Vacutainer blood collection tubes with sodium heparin. Mononuclear cells were then isolated using a sterile density gradient centrifugation with Ficoll-Paque PLUS (density 1.077 g/mL) according to the manufacturer's instructions. PBMC cell suspension was adjusted to a 2 x 10^6 cells/mL in RPMI 1640 containing 20% autologous serum. The cell suspension was transferred into 350 mL perfluoroalkoxy alkane (PFA) jars (Savillex Corporation, Eden Prairie, MN) and closed loosely with screw caps. The jars were placed into a 5% CO_2 incubator at 37°C for five days, during which PBMCs differentiated into macrophages. Next, the cells were washed with cold RPMI 1640 three times via centrifugation at 300 g for 10 minutes and 4°C. The cells were counted and seeded onto plates at indicated densities using RPMI 1640 with 10-20% autologous serum and 50 ng/mL human macrophage colony stimulating factor (M-CSF, Miltenyi Biotec) to allow for complete differentiation. The experiments were performed on day seven of differentiation.

Primary mouse carotid artery endothelial cells

Primary mouse carotid artery endothelial cells were purchased from Cell Biologics Inc. (Chicago, IL) and cultured using complete mouse endothelial cell medium (Cell Biologics Inc.) on gelatin-coated dishes and plates and according to the vendor's recommendations.

Quantitative real-time PCR

Total RNA was extracted from cells using the Absolutely RNA Miniprep Kit (Agilent-Stratagene) according to the manufacturer's protocol, and treated with DNase I to remove residual DNA. Total RNA from tissue was extracted using TRIzol (Thermo Fisher). RNA from magnetically-separated cells was extracted using the Absolutely RNA nanoprep kit (Agilent Technologies). Total RNA (>0.5 µg) was reverse-transcribed and cDNA was amplified by PCR using the High Capacity cDNA Reverse Transcription Kit from Thermo Fisher. The sequences of the primers and TaqMan probes are listed in Supplementary Table S1. Gene expression was quantified using the Roche LightCycler 480 detection system. Amplification was detected with SYBR Green as a fluorogenic probe specific for double-stranded DNA by using a Power SYBR Green PCR Master Mix (Roche Diagnostics GmbH). Gene amplification was normalized to 2-3 different housekeeping genes and the best stable control genes were identified using geNorm.[13] Relative copy number (RCN) and fold change were determined. RCN was

calculated as follows: RCN = E-ΔCt × 100, where E is the efficiency (E = 10^[-1/slope] 2.0 = 100% efficient) and ΔCt = Ct(target) - Ct(reference). Duplicate samples were analyzed in each experiment.

Oral glucose tolerance test (OGTT) and glycemia metrics

Mice were fasted with full access to water for 16 hours before oral gavage with glucose at 2 g per kilogram body weight. Mice were not anesthetized during gavage. Nanoparticles or liraglutide were injected 2 hours prior to OGTT. Fasting blood glucose was measured using a hand-held glucometer (Ascensia Diabetes Care, Parsippany, NJ) from saphenous vein blood collected before gavage and re-sampled every 5-10 minutes for two hours after the gavage. Approximately 20 µL of blood was collected at every time point into an EDTA capillary tubes for the analysis of blood insulin concentrations using ultra-sensitive enzyme-linked immunosorbent assay (ELISA, Crystal Chem).

Flow cytometry, fluorescence-activated cell sorting (FACS)

Single-cell suspensions from an aortic sample or whole blood were prepared as previously described.[4] Briefly, perfused aortas were cut lengthwise and finely minced in <2 mm pieces in a well of a 6-well plate. The pre-warmed enzyme cocktail consisting of liberase (4 U/mL), DNAse I (50 U/mL) and hyaluronidase (60 U/mL) in 2 mL of serum-free RPMI was then added and incubated for 1 h at 37°C. The pieces of semi-digested tissue were then agitated with pipetting using a large-orifice pipette tip and additionally incubated for 30 min. The mixture was then added to a 10 mL of cold RPMI containing 5% FBS and centrifuged (200 g x 10 min, 4°C). The cell pellets were resuspended in RPMI+ 5% FBS and passed through a 50 µm cell strainer before repeating the centrifugation step. Cell suspensions containing 10^6 cells were put in 1.5 ml centrifuge tubes and diluted to 1 ml. The tubes were centrifuged (400 g, 5 min, 4°C) and all but 100 µl of supernatant was carefully removed. The cells were resuspended by gentle tapping and then 1-3 µl of each FACS antibody was added. Cells were incubated for 20 min at 4°C with gentle tapping every 5 min. Next, 1 mL of FACS buffer (1% FBS in DPBS) was added and tubes were centrifuged (400 g, 5 min, 4°C). The cells were washed twice by resuspending in the FACS buffer and centrifuging. Finally, cells were fixed in 0.5 mL of 1% phosphate buffered formalin and kept at 4 ^{o}C until analysis by flow-cytometry using Becton Dickinson LSR II equipped with 488 nm blue and 633 nm red lasers and the following mirrors/filters: 780/60, 670/14, 610/20, 575/26, 530/30, 488/10, 730/45, 660/20. See Supplementary Table S1 for antibody details.

Lipid analysis

Plasma lipids were analyzed using fast protein liquid chromatography (FPLC). The FPLC system consisted of the Gilson GX271 Liquid Handler (Gilson Inc, Middleton, WI) equipped with a PHD ULTRA HPSI/XF high precision, high pressure syringe pump (Harvard Apparatus, Holliston, MA). Separation of lipoproteins was performed on a Superose 6 Increase 3.2/300 column (GE Healthcare Bio-Sciences, Pittsburgh, PA). Plasma samples were diluted ten times

with a phosphate buffered saline followed by an injection into the column using a Gilson GX Direct Injection Module equipped with a 10 µL injection loop. The reproducibility of the separation was achieved using “loop volume overfill” task, which overfilled the loop with a 40 µL of the sample before each injection. Lipoproteins were eluted in an isocratic mode with a buffer containing 10 mM phosphate and 154 mM NaCl at pH 7.4 and room temperature. The flow rate was 0.05 mL/min. Fifty 100 µL fractions were collected in opaque 96-well plates positioned on a cooling block at 4 °C. The fractions were then analyzed for cholesterol and triglyceride concentrations using hydrogen peroxide detection assays based on the peroxidase-oxidase reaction as previously described[14,15] and briefly outlined below.

Plasma samples or FPLC fractions were incubated with cholesterol or triglyceride reagents that were prepared from fresh lyophilized enzymes on the day of analysis as follows (see Supplementary Table S1 for enzyme details). The cholesterol reagent consisted of 25 mM PIPES at pH 6.7, 250 U/mL cholesterol oxidase, 1000 U/mL cholesterol esterase, 1 U/mL peroxidase (horseradish), 5 mM sodium taurocholate, and 0.25 mg/mL Amplex Red (10-Acetyl-3,7-dihydroxyphenoxazine). The triglyceride reagent was prepared in 25 mM PIPES at pH 6.7 with 2.5 mM ATP, 2.5 mM magnesium acetate, 10 U/mL glycerol 3-phosphate oxidase, 5 U/mL glycerokinase, 100 U/mL lipase, 1 U/mL peroxidase (horseradish), and 0.25 mg/mL Amplex Red. Fifty microliters of the sample (FPLC fraction or 10x diluted plasma sample) was incubated with 150 µL of either cholesterol or triglyceride reagent in opaque 96-well plate along with the appropriate clinical-grade standards at a range of concentrations of 1 - 1000 mg/dL. The standards were from Stanbio Laboratory (Boerne, TX) or Pointe Scientific (Canton, MI) as indicated in the Supplementary Table S1. The plate was incubated at 37°C for 30 min and the Amplex Red fluorescence was recorded at 544 nm excitation and 594 nm emission using SpectraMax i3 plate reader (Molecular Devices, San Jose, CA). The concentration of cholesterol or triglycerides was derived from the respective standard curves using SoftMax Pro 6 software and plotted against FPLC fraction number. The total concentration of lipids in vLDL, LDL and HDL fractions were determined by the integration of the FPLC peaks and obtaining the area under the curve.

Liraglutide and GlpNP proteolytic stability assays

Liraglutide or GlpNP were resuspended at 0.02 mg/mL (based on liraglutide concentration) in an incubation buffer consisting of 20 mM HEPES buffered saline pH 7.4, 1 mM magnesium chloride, 1.5 mM zinc chloride and 1% pooled human plasma (Red Cross). 90 µL of each solution was transferred to a vial equipped with a magnetic stir bar and containing 10 µL of 0.4 mg/mL CHO-expressed Neprilysin (Bio-Techne Corporation, Minneapolis, MN). In some experiments, recombinant MMP2 (1 µg/mL, Bio-Techne) plus MMP9 (1 µg/mL, Bio-Techne) were used. The solution was stirred at 37°C and 5 µL aliquots were taken at predetermined time points (See Figure 2B). The aliquots were then quenched with 45 µL of 1% trifluoroacetic acid (TFA) in 80% acetonitrile / 20% water. The quenched solutions were transferred in 0.45 µm PTFE mini prep filter vials (Analytical Sales and Services, Flanders, NJ) and filtered. Next, the concentration of liraglutide was determined using high performance liquid chromatography (HPLC) as described below. In gelatinase degradation experiments, aliquots were not taken, but rather 10 µL of GlpNP particles were diluted in 0.5 mL PBS and spun down at 100,000 g (10

min, 4°C). The supernatant was aspirated by vacuum suction and the nanoparticle pellet was processed in TFA/acetonitrile/water as above.

The separation was performed using the Shimadzu Prominence system consisting of three LC-20AD pumps, autosampler, RF-20Axs fluorescence detector and Sedex 90LT evaporative light scattering detector (ELSD). The sensitive detection (detection limit >2 pmol of liraglutide) was possible due to intrinsic fluorescence of liraglutide due to the presence of tryptophan in the peptide sequence, which was detected at 280 nm excitation and 350 nm emission wavelengths respectively. Five microliters were injected into 4.6 mm x 50 mm Promix MP column (SIELC Technologies, Wheeling, IL), which was equilibrated and maintained at 40°C using a column heater. The mobile phase was delivered at 1 mL/min using three pumps: A 100% water, B 100% acetonitrile and C 1% TFA. The gradient was formed by pumping from 15% B to 45% B over 25 min, while concentration of TFA was maintained constant at 10% C (0.1% of TFA total). The RF-20Axs peaks were manually integrated using Shimadzu LabSolutions software and the data were expressed as a percent of the liraglutide peak area at time 0 (initial concentration). The peaks from the ELSD detector were not used for quantification and served as a qualitative assessment of the formation of low molecular weight degradation products that are not possible to register with the UV or fluorescence detector.

Magnetic resonance imaging (MRI)

MRI of atherosclerotic plaque in Apoe$^{-/-}$ mice injected with gadolinium(Gd)-containing GlpNP was performed as previously described.[4,6,16] In brief, 9.4T small bore animal imaging magnet Bruker BioSpec was used to acquire gradient echo sequences (GRE) in 3-5 mice per group (GlpNP, CtrNP, PtdSer^{neg}CtrlNP) pre and 24 h post NP injection. Animals were administered with a 300 µL of NP formulation at 0.05 mg/kg of Gd. Localization acquisition parameters were as follows: 0.1×0.1×0.5 mm^3 transversal contiguous slices, TR=316, TE=3.7 ms, FA=20°, four averages; GRE parameters were: TR=28ms, TE=4.9-10ms, FA=30°, four averages, resolution=0.095 mm^2. Twenty four slices were acquired from renal artery to bifurcation. Images were analyzed by OsiriX software available free of charge at www.osirix-viewer.com. Contrast-to-noise ratio (CNR) was calculated as CNR=[signal intensity(plaque) - signal intensity(muscle)]/standard deviation(noise). Abbreviations: TR-Repetition Time; TE-echo time; FA-flip angle. Mice were euthanized as described above in the Animals section.

Synthesis of acetylated LDL and BODIPY-cholesterol-acetylated LDL

Acetylated LDL was prepared as previously described.[17] Briefly, 1 mL of 10 mg/mL of LDL was combined with 1 mL saturated sodium acetate solution and the mixture was stirred on ice at 200 rpm using a magnetic stir bar. Acetic anhydride was then added in 2 µL portions and 10 min intervals to a final concentration of 6 µL acetic anhydride per 1 mg LDL protein. The acetylated lipoprotein solution was dialyzed against 10 mM phosphate buffer plus 154 mM NaCl. Next, 50 µL of 50 mg/mL BODIPY cholesterol (TopFluor cholesterol, Avanti Polar Lipids) in 2-propanol was added to 1 mL lipoprotein deficient serum and stirred at 37°C for 4 h. Five milliliters of acetylated LDL at 1 mg/mL was then added to the above mixture and incubated at 37°C

overnight with magnetic stirring. BODIPY-cholesterol-acetylated LDL was then isolated by ultracentrifugal flotation (4 h, 400,000 g at 4°C) with the density adjusted to 1.063 g/L. The centrifugation was performed using Thermo Scientific Sorvall MX 120 Plus ultracentrifuge and S120-AT3 rotor. The product was collected as a top band visible in UV light. The final product was dialyzed overnight against 10 mM phosphate buffered saline at 4°C and filtered through a 0.45 µm cellulose acetate membrane using a syringe filter before treating the cells

Cholesterol efflux studies

Human primary aortic smooth muscle cells (HASMC) were purchased from ATCC (PCS-100-012) and cultured in Vascular Cell Basal Medium (ATCC PCS-100-030) supplemented with Vascular Smooth Muscle Cell Growth Kit (ATCC PCS-100-042). The cells were seeded onto opaque 96-well plates at 2×10^5 cells per well and cultured in 100 µL of medium at 37°C in a 5% CO_2 atmosphere for 24 h. Next, HASMC were cholesterol-loaded by incubation with 100 µg/mL BODIPY-cholesterol-acetylated LDL (obtained as described above) in the serum-free medium containing 1.5% human serum albumin for 48 hours. One day prior to the cholesterol efflux experiments, the cells were incubated for 24 hours in the medium containing 1.5% human serum albumin and in the presence or absence of 10 nM liraglutide, GlpNP (containing 10 nM liraglutide and 0.1 mg/mL polystyrene nanoparticles), CtrlNP (0.1 mg/mL polystyrene nanoparticles), and medium alone. In some experiments, 1 hour prior to incubation with nanoparticles, 1 µM cAMP inhibitor (3-Isobutyl-1-methylxanthine) or 100 nM GLP-1R antagonist (exendin fragment 9-39) or 10 µM forskolin was added. Next, the medium was removed and replaced with 100 µL of fresh culture medium containing only Vascular Cell Basal Medium and 1.5% human serum albumin. This was repeated twice over the next 1 hour. Then, these BODIPY-cholesterol-acetylated LDL-loaded HASMCs were incubated in 100 µL serum-free medium with or without apoA-I (50 µg/mL) or HDL (100 µg/mL) for 24 hours. The fluorescence at excitation of 490 nm and emission of 515 nm of the plates was recorded. The result of this reading is Total Cell Fluorescence (TCFI). The efflux medium was then transferred to a new opaque 96-well plate (or to the empty wells of the same plate) with a multichannel pipette. Fluorescence at excitation of 490 nm and emission of 515 nm was read, resulting in Efflux Medium Fluorescence (EMFI). The cholesterol efflux was calculated as: % Efflux = 100 x [EMFI] / [TCFI]. The formula used was Net Fluorescence = [Total Fluorescence] - [Background Fluorescence]. Background Fluorescence was measured in wells that did not have BODIPY.

RNA sequencing

For bulk RNA-seq, samples were lysed with Trizol reagent and the RNA was extracted and purified as described above. Full length RNA-seq libraries were prepared by Novogene corporation and sequenced using Illumina HiSeq platform. Raw data were recorded in an FASTQ file and analyzed as previously described.[18] The expression information on specific genes of interest was extracted from normalized data (RPKM) using R version 3.6.0 and dplyr package and plotted against normalized densitometry data from Southern blotting experiments (see above). All data associated with this study have been deposited in the Sequence Read Archive (SRA) at NCBI under the BioProject IDs: PRJNA544396 and PRJNA678544.

Atherosclerosis studies and analysis

Morphological analysis of atherosclerotic plaque and immunohistochemistry was performed as previously described.[19] The stained slides were imaged using Keyence BZ-X700 microscope and analyzed using Keyence BZ-X Analyzer software ver. BZ-H3XD. Hybrid cell count mode was used to determine areas of plaque, necrotic core or F4/80 (or αSMA)-positive cells. Next, the selection was made using Extraction by Brightness mode and the threshold was set at 864. Unwanted areas were removed by filtering by area and shape. Modes such as "fill cracks" and "deburring" were further used to refine the selection. BZ-X Analyzer output was area in μm^2 or mm^2 and number of total areas. The data were analyzed using repeated measures ANOVA with Shapiro-Wilk test of normality and 3-way between-subjects ANOVA using fixed-effects model. Equality of variances was tested using the Bonferroni-Dunn post hoc test.

Myography

Myographic analysis of the isolated aortic rings was performed as previously described.[20,21] Briefly, mice were anesthetized and the thoracic aorta was removed, cleaned and placed in physiological salt solution (PSS) containing: 130 mM NaCl, 14.9 mM $NaHCO_3$, 4.7 mM KCl, 1.18 mM KH_2PO4, 1.18 mM $MgSO_4$, 1.56 mM $CaCl_2$, 0.026 mM EDTA, and 5.5 mM glucose. The tissue was cut into 2-mm rings that were mounted in a chamber of a Multi Myography System (Danish Myo Technology A/S) containing PSS at 37°C and continuously aerated with 95% O_2-5% CO_2. Isometric force generation was recorded with a resting tension of 30 mN, and the rings were allowed to equilibrate for 2 h. Arterial integrity was assessed first by stimulation of vessels with 80 mM KCl. Endothelium-integrity was assessed by measuring the dilatory response to 10 μM acetylcholine in vessels pre-contracted with 1 μM phenylephrine. To test aortic relaxation, the rings were pre-contracted with 0.3 μM phenylephrine that induced approximately 60% of maximal contraction, which was followed by addition of acetylcholine (ACh) in a cumulative manner and at the following doses in nM: 1, 3, 10, 30, 100, 300, 1000.

Cholesterol analysis in atherosclerotic plaque

Free cholesterol concentration in atherosclerotic plaque was obtained after extraction, derivatization with pentafluorophenyl isocyanate (PFPI) and analysis by liquid chromatography/mass spectrometry (LCMS) as previously described.[19,22,23] Briefly, excised atherosclerotic plaques were homogenized in 1 ml of hexane-isopropanol 2:1 (v/v) with 1 nM cholesterol-d_7 added as an internal standard. The extraction was repeated with obtained precipitates two more times and combined extracts were centrifuged, filtered and evaporated under a stream of argon gas. The residue was re-dissolved in 1 mL 100% hexane and 3 μL pyridine and 5 μL of PFPI was added. After 10 min at room temperature, 100 μL of 100% isopropanol was added to quench the reaction and dissolve the precipitate.

Five microliters of derivatized extracts were injected into Agilent Zorbax Rx-SIL column (150 x 4.6 mm, 5 μm) and separated using Shimadzu Prominence HPLC (see above) using an

isocratic solvent system consisting of 90/10 hexane/isopropanol and a flow rate of 1 mL/min. The effluent was analyzed on the AB Sciex 3200 QTRAP LC/MS/MS system coupled with an atmosphere pressure chemical ionization (APCI) ionization mode. Mass spectra were acquired in negative ion mode using a capillary temperature set at 300°C, and spray voltage was set at 3.5 kV. Nebulizer gas and auxiliary gas flow were set to 60 and 20 arbitrary units respectively. Curtain gas (CUR) was set to 15.00 and collision gas (CAD) settings were set to medium. Detection was by multiple reaction monitoring (MRM) with the transitions monitored 595.4→206.0, 602.5→206.0 corresponding to cholesterol and cholesterol-d_7 respectively. Total cholesterol was analyzed after hydrolysis of cholesteryl esters with sodium methoxide as previously described,[22] levels were analyzed as described above.

Statistical analysis

We examined an N of 6-12 per group for cell-based *in vitro* assays and indicated in figures N for each group for mouse experiments. Data were tested for normality using the D'Agostino-Pearson normality test and for equality of variances using Bartlett's test. If normality and equality of variances are not rejected at the significance level of 0.05, the group means were compared using Student’s t-test (for 2 groups) or pairwise t-test (for >2 groups) with Holm *post hoc* correction or ANOVA with Tukey’s test (for >2 groups). For non-normally distributed data or data with unequal variances, nonparametric Mann-Whitney U-test (for 2 groups) and Kruskal-Wallis test and Dunn’s test (for >2 groups) all with Holm *post hoc* correction.

Supplementary Table S1. Resources table: reagents, materials and supplies used in this work

**Reagent or Resource**	**Source**	**Identifier or catalog number**
Monoclonal anti-human GLP1R antibody (Mab 3F52)	Novo Nordisk	MAb 3F52
Monoclonal anti-mouse GLP1R antibody (Mab 7F38)	Developmental Studies Hybridoma Bank	Mab 7F38
Liraglutide	Bachem Americas	H6724.0005
Exendin Fragment 9-39	Sigma-Aldrich	E7269
Neprilysin (CD10)	Bio-Techne Corporation	1182ZNC010
Lipoproteins, Low Density, HP, Cell Culture	Athens Research and Technology	12-16-120412-TC
Lipoproteins, Oxidized LDL (OX-LDL), Human Plasma	Athens Research and Technology	12-16-120412-OX
CP-113,818 (ACATi)	Pfizer	CP-113,818 (through MTA)
Lipoproteins, High Density, HP, Cell Culture	Athens Research and Technology	12-16-080412-TC
Cholesterol-Water Soluble	Sigma	C4951
Carboxyl latex, 4% w/v 0.04 μm	Life Technologies	C37262
Carboxyl latex, 2.5% w/v 2 μm	Polysciences Inc.	18327-10
Avidin Polystyrene particles, crosslinked, 0.5% w/v 7.23 um	Spherotech	VPX-60-5
Gelatin from porcine skin, Type A	Sigma-Aldrich	G2625
DSPE-PEG(2000) Carboxylic Acid	Avanti Polar Lipids	880135
1-palmitoyl-2-oleoyl-sn-glycero-3-phospho-L-serine	Avanti Polar Lipids	840034
TopFluor (BODIPY) Cholesterol	Avanti Polar Lipids	810255
Cholesterol oxidase (microbial)	Sigma-Aldrich	C1235
Cholesterol Esterase (microbial)	EMD Millipore	228180
Peroxidase (Horseradish)	Sigma	SRE0082
Aqua Block	Eastcoast Bio	Cat#PP82
Sodium taurocholate	Sigma	86339
Adenosine 5′-triphosphate (ATP) disodium salt hydrate	Sigma	FLAAS
Glycerol 3-phosphate Oxidase from *Pediococcus* sp.	Sigma	G9637
Glycerokinase from *Cellulomonas* sp.	Sigma	G6142
Lipase from *Pseudomonas* sp.	Sigma	L9518

10-Acetyl-3,7-dihydroxyphenoxazine	Sigma	90101
Cholesterol Standard (200 mg/dL)	Stanbio Laboratory	1012-030
Triglycerides Standard 2 mg/mL	Pointe Scientific	T7531-STD
Arg1 RT-PCR assay	Thermo Fisher	Mm00475988_m1
Tubb3 RT-PCR assay	Thermo Fisher	Mm00727586_s1
Nos2 RT-PCR assay	Thermo Fisher	Mm00440502_m1
Il1b RT-PCR assay	Thermo Fisher	Mm00434229_m1
Ccl2 RT-PCR assay	Thermo Fisher	Mm00441242_m1
Vcam1 RT-PCR assay	Thermo Fisher	Mm01320970_m1
Nr1h3 RT-PCR assay	Thermo Fisher	Mm00443451_m1
Abca1 RT-PCR assay	Thermo Fisher	Mm00442646_m1
Abcg1 RT-PCR assay	Thermo Fisher	Mm00437390_m1
Abcg5 RT-PCR assay	Thermo Fisher	Mm00446241_m1
Abcg8 RT-PCR assay	Thermo Fisher	Mm00445980_m1
Lrp1 RT-PCR assay	Thermo Fisher	Mm00464608_m1
Scarb1 RT-PCR assay	Thermo Fisher	Mm00450234_m1
Lipc RT-PCR assay	Thermo Fisher	Mm00433979_m1
Cpt1a RT-PCR assay	Thermo Fisher	Mm01231183_m1
Fabp1 RT-PCR assay	Thermo Fisher	Mm00444340_m1
Fabp2 RT-PCR assay	Thermo Fisher	Mm00433188_m1
Plin1 RT-PCR assay	Thermo Fisher	Mm00558672_m1
Apob RT-PCR assay	Thermo Fisher	Mm01545150_m1
Gapdh RT-PCR assay	Thermo Fisher	Mm99999915_g1
Mrc1 RT-PCR assay	Thermo Fisher	Mm01329632_m1
Hprt RT-PCR assay	Thermo Fisher	Mm00446969_m1
Glp1r RT-PCR assay	Thermo Fisher	Mm01351008_m1
B4galt1 RT-PCR assay	Thermo Fisher	Mm00480752_m1
Actb RT-PCR assay	Thermo Fisher	Mm00607939_s1
TNF RT-PCR assay	Thermo Fisher	Mm00443258_m1
IL6 RT-PCR assay	Thermo Fisher	Mm00446190_m1
Human GLP1R RT-PCR assay	Thermo Fisher	Hs00157705_m1
Human ACTB RT-PCR assay	Thermo Fisher	Hs01060665_g1
Human RPL37A RT-PCR assay	Thermo Fisher	Hs01102345_m1

Human Bactin F	Eurofins Scientific	ATGATATCGCCGCGCTCGTC
Human Bactin R	Eurofins Scientific	CGCTCGGTGAGGATCTTCA
Human glp1r (a) F	Eurofins Scientific	CTACGCACTCTCCTTCTCTGCT
Human glp1r (a) R	Eurofins Scientific	CGGACAATGCTCGCAGGATGAA
Human glp1r (b) F	Eurofins Scientific	CTGCTGTTTGTTGTCCCCT
Human glp1r (b) R	Eurofins Scientific	AGAATGGGCAGCCGGATAA
Mouse glp1r (southern blotting) F	Integrated DNA Technologies	GTACCACGGTGTCCCTCTCA
Mouse glp1r (southern blotting) R	Integrated DNA Technologies	CCTGTGTCCTTCACCTTCCCTA
Mouse glp1r DIG probe (southern blotting) internal hybridization probe	Integrated DNA Technologies	5'GCTGTATCTGAGCATAGGCT/3Dig_N/-3'
Mouse Hprt (southern blotting) F	Integrated DNA Technologies	ACCTCTCGAAGTGTTGGATACAG
Mouse Hprt (southern blotting) R	Integrated DNA Technologies	TTCACTAATGACACAAACGTGATTC
Platinum SuperFi II DNA Polymerase	Thermo Fisher	12368050
SuperScript IV Reverse Transcriptase	Thermo Fisher	18091200
DIG Wash and Block Buffer Set	Roche/Sigma-Aldrich	11585762001
CDP-Star Chemiluminescent Substrate	Sigma-Aldrich	C0712
High Capacity cDNA Reverse Transcription Kit	Thermo Fisher	4368814
Liberase ™ Research Grade	Roche/Sigma-Aldrich	05401127001
DNase I recombinant	Roche/Sigma-Aldrich	04716728001
Hyaluronidase	Sigma-Aldrich	H3884
Rat monoclonal [CI:A3-1] to F4/80	Abcam	ab6640
Mouse monoclonal [KP1] to CD68	Abcam	ab955
Rabbit monoclonal [EPR6573] to CD36	Abcam	ab133625
Anti-Digoxigenin-AP, Fab fragments	Roche/Sigma-Aldrich	11093274910
Monoclonal Anti-Actin, α-Smooth Muscle (αSMA)	Sigma-Aldrich	A2547
Goat polyclonal Anti-Actin, α-Smooth Muscle (αSMA)	Sigma-Aldrich	SAB2500963
Transgelin/SM22	Proteintech	10493-1-AP
SMMHC	Proteintech	21404-1-AP
CD107b (Mac3)	BD Biosciences	550292
Galectin-3	Santa Cruz Biotechnology	sc-23938
CD11b APC-Cy7 Rat anti-Mouse	BD Biosciences	561039

IgG2bκ M1/70		
CD11c APC Armenian Hamster anti-Mouse IgG1 λ2 HL3	BD Biosciences	561119
Ly-6C eFluor 450 Rat anti-Mouse IgG2cκ HK1.4	eBioscience (Thermo Fisher Scientific)	48-5932-80
CD36 Monoclonal Antibody (MF3), FITC	eBioscience (Thermo Fisher Scientific)	MA5-16832
PE Rat Anti-Mouse CD181 (CXCR1) Clone U45-632	BD Biosciences	566383
anti-rat IgG Alexa Fluor 594	Thermo Fisher	A11007
goat anti-mouse IgG Alexa Fluor Plus 488	Thermo Fisher	A32742
Goat anti-Mouse IgG2a Cross-Adsorbed Secondary Antibody, Alexa Fluor 647 (for mouse GLP1R mAb 7F38 detection)	Thermo Fisher	A-21241
goat anti-rabbit IgG Alexa Fluor Plus 594	Thermo Fisher	A32740
Donkey anti-Goat IgG (H+L) Highly Cross-Adsorbed Secondary Antibody, Alexa Fluor Plus 488	Thermo Fisher	A32814TR
Mouse IgG2a Isotype Control (isotype control for mouse GLP1R mAb 7F38 detection)	Thermo Fisher	PA5-33241
Mouse IgG1 Isotype Control (isotype control for human GLP1R mAb 7F38 detection)	Thermo Fisher	PA5-33198
APC Annexin V	BioLegend	640920
CD11b MicroBeads	Miltenyi Biotec Inc.	130-049-601
CD11c MicroBeads UltraPure	Miltenyi Biotec Inc.	130-108-338
Human M-CSF, premium grade	Miltenyi Biotec Inc.	130-096-492
4.6 mm x 50 mm Promix MP HPLC column	SIELC Technologies	MP-46.050.0530
Superose 6 Increase 3.2/300 FPLC	GE Healthcare Bio-Sciences	29091598
Ultra-sensitive mouse insulin ELISA	Crystalchem	90080
µ-Slide 8 Well chambered coverslip	ibidi GmbH	80826
Cyclic AMP ELISA Kit	Cayman Chemical	581001
Mouse Apolipoprotein B48 ELISA Kit	MyBioSource, Inc.	MBS744267
Ficoll-Paque PLUS	GE Healthcare Bio-Sciences	17-1440-02
Mice B6.129P2-Apoe$^{tm1Unc/J}$	The Jackson Laboratory	Stock No: 002052
High fat rodent diet with 1.25% cholesterol	Research Diets	Product # D12108C

Pentafluorophenyl isocyanate	Sigma Aldrich	512079

Supplementary Table S2: Screening of different nanoparticle formulations by varying concentrations of gelatin, PEG, and the core (polystyrene nanoparticles). The criteria for the "best" formulation were a) the smallest hydrodynamic diameter, b) the lowest polydispersity, and c) highest liraglutide incorporation. Five "lead" formulations are shown out of 32 different formulations screened (data not shown). Highlighted in yellow is the GlpNP used in this paper.

	**Control**	**G3P3 (GlpNP)**	**G3P4**	**G3P5**	**GC3P3**	**GC3P4**	**GC3P5**
Gelatin (mg/mL)	0	0.28672	0.28672	0.28672	2.8672	2.8672	2.8672
PEG Concentration (mg/mL)	0	5	10	20	5	10	20
Core Concentration (mg/mL)	0.002	0.002	0.002	0.002	0.002	0.002	0.002
Diameter (nm)	49.8	53.7	52.6	54.9	76.8	69.7	65.6
Polydispersity	0.114	0.083	0.083	0.137	0.132	0.136	0.186
Zeta Potential (mV)	-29.242	-11.85	-24.096	-17.56	-3.48	-5.608	-14.806
Incorporation Efficiency, %	NA	41.67	10.12	12.26	NA	NA	NA
Liraglutide (mg/mL)	not detected	0.35	0.085	0.103	not detected	not detected	not detected

Supplementary Table S3: Results of the correlation analysis between *Glp1r* expression by Southern blotting (see Figure 1M of main manuscript) and the levels of various myeloid and smooth muscle-related gene mRNA transcripts (as indicated) determined by RNAseq. Correlation values are shown as R^2 and p values were determined from multiple comparisons of means (ANOVA with Tukey's post-hoc test).

	**Smooth muscle cell markers**											
	**Acta2**	**Cald1**	**Cdh5**	**Cnn1**	**Hexim1**	**Hrh2**	**Myh11**	**Tpm1**	**Tagln**	**Klf2**	**Klf4**	**Klf5**
**R^2**	0.0672	0.1246	0.1412	0.0435	0.1798	0.0824	0.0239	0.1040	0.0560	0.1556	0.1815	0.0016
**p-value**	0.2214	0.0906	0.0704	0.3283	0.0389	0.1739	0.4706	0.1242	0.2655	0.0564	0.0379	0.8521
	**Macrophage/ myeloid cell markers**											
	**Cd68**	**Lamp2**	**Lgals3**	**S100a9**	**Cd163**	**Cd80**	**Cd206 (Mrc1)**	**CD11b**	**CD11c**	**CD3**	**S100a9**	
**R^2**	0.1526	0.3741	0.1507	0.0137	0.02238	0.1818	0.3100	0.0426	0.0461	0.0545	0.0077	
**p-value**	0.0591	0.0015	0.0609	0.5859	0.4853	0.0377	0.0047	0.4115	0.3922	0.3512	0.7297	

Legend:	Not significant, but low p value
	Significant
	Not significant

Supplementary Table S4

P values calculated from qPCR analysis of indicated genes in support of main manuscript Figure 2B

		ctr	ACATi	Chol	Chol+ACATi	Lira	Lira + Chol +ACATi
ACATi	Acta2	0.246	NA	NA	NA	NA	NA
Chol		0.187	1.000	NA	NA	NA	NA
Chol+ACATi		0.000	0.054	0.067	NA	NA	NA
Lira		0.246	1.000	1.000	0.054	NA	NA
Lira + Chol +ACATi		0.246	1.000	1.000	0.054	1.000	NA
2 µm Latex beads		1.000	0.603	0.481	0.001	0.603	0.603
ACATi	Tpm1	1.000	NA	NA	NA	NA	NA
Chol		1.000	1.000	NA	NA	NA	NA
Chol+ACATi		0.887	0.887	1.000	NA	NA	NA
Lira		1.000	1.000	1.000	1.000	NA	NA
Lira + Chol +ACATi		0.466	0.466	1.000	1.000	1.000	NA
2 µm Latex beads		1.000	1.000	1.000	1.000	1.000	1.000
ACATi	Myh11	1.000	NA	NA	NA	NA	NA
Chol		1.000	1.000	NA	NA	NA	NA
Chol+ACATi		0.053	1.000	0.365	NA	NA	NA
Lira		1.000	1.000	1.000	0.148	NA	NA
Lira + Chol +ACATi		0.333	1.000	1.000	1.000	0.787	NA
2 µm Latex beads		1.000	1.000	1.000	0.562	1.000	1.000
ACATi	Hexim1	1.000	NA	NA	NA	NA	NA
Chol		0.232	1.000	NA	NA	NA	NA
Chol+ACATi		1.000	1.000	1.000	NA	NA	NA
Lira		0.081	0.399	1.000	0.553	NA	NA
Lira + Chol +ACATi		0.232	1.000	1.000	1.000	1.000	NA
2 µm Latex beads		0.029	0.176	1.000	0.232	1.000	1.000
ACATi	Cnn1	0.000	NA	NA	NA	NA	NA
Chol		0.005	0.000	NA	NA	NA	NA
Chol+ACATi		0.000	0.000	0.000	NA	NA	NA
Lira		0.000	0.030	0.000	0.000	NA	NA
Lira + Chol +ACATi		0.000	0.145	0.000	0.000	0.003	NA
2 µm Latex beads		0.000	0.000	0.096	0.000	0.011	0.000
ACATi	Klf4	1.000	NA	NA	NA	NA	NA
Chol		1.000	1.000	NA	NA	NA	NA
Chol+ACATi		0.080	0.035	0.326	NA	NA	NA
Lira		1.000	1.000	1.000	0.015	NA	NA
Lira + Chol +ACATi		1.000	1.000	1.000	0.310	1.000	NA
2 µm Latex beads		0.384	0.821	0.096	0.001	1.000	0.102
ACATi	Tagln	0.368	NA	NA	NA	NA	NA
Chol		1.000	0.461	NA	NA	NA	NA

Chol+ACATi		1.000	1.000	1.000	NA	NA	NA
Lira		1.000	1.000	1.000	1.000	NA	NA
Lira + Chol +ACATi		1.000	1.000	1.000	1.000	1.000	NA
2 µm Latex beads		1.000	1.000	1.000	1.000	1.000	1.000
ACATi	Lamp2	1.000	NA	NA	NA	NA	NA
Chol		1.000	1.000	NA	NA	NA	NA
Chol+ACATi		0.070	0.169	0.087	NA	NA	NA
Lira		1.000	1.000	1.000	0.169	NA	NA
Lira + Chol +ACATi		1.000	1.000	1.000	0.657	1.000	NA
2 µm Latex beads		1.000	1.000	1.000	0.087	1.000	1.000
ACATi	Lgals3	0.308	NA	NA	NA	NA	NA
Chol		0.043	0.905	NA	NA	NA	NA
Chol+ACATi		0.000	0.000	0.000	NA	NA	NA
Lira		0.905	0.215	0.029	0.000	NA	NA
Lira + Chol +ACATi		0.000	0.000	0.000	0.000	0.000	NA
2 µm Latex beads		0.905	0.905	0.323	0.000	0.745	0.000
ACATi	Abca1	1.000	NA	NA	NA	NA	NA
Chol		0.000	0.000	NA	NA	NA	NA
Chol+ACATi		0.000	0.000	0.000	NA	NA	NA
Lira		1.000	1.000	0.000	0.000	NA	NA
Lira + Chol +ACATi		0.000	0.000	0.000	0.025	0.000	NA
2 µm Latex beads		1.000	1.000	0.000	0.000	1.000	0.000
ACATi	Abcg1	1.000	NA	NA	NA	NA	NA
Chol		0.000	0.000	NA	NA	NA	NA
Chol+ACATi		0.000	0.000	0.142	NA	NA	NA
Lira		1.000	1.000	0.000	0.000	NA	NA
Lira + Chol +ACATi		0.000	0.000	0.142	1.000	0.000	NA
2 µm Latex beads		1.000	1.000	0.000	0.000	1.000	0.000
ACATi	Hmox1	1.000	NA	NA	NA	NA	NA
Chol		1.000	1.000	NA	NA	NA	NA
Chol+ACATi		0.000	0.000	0.000	NA	NA	NA
Lira		0.065	0.024	0.011	0.028	NA	NA
Lira + Chol +ACATi		1.000	0.848	0.433	0.001	0.296	NA
2 µm Latex beads		0.030	0.011	0.005	0.061	1.000	0.132

References to Supplementary Methods

1. Pyke C, Heller RS, Kirk RK, et al. GLP-1 receptor localization in monkey and human tissue: novel distribution revealed with extensively validated monoclonal antibody. Endocrinology. 2014; 155: 1280–90.
2. Kampfrath T, Maiseyeu A, Ying Z, et al. Chronic fine particulate matter exposure induces systemic vascular dysfunction via NADPH oxidase and TLR4 pathways. Circ Res. 2011; 108: 716–26.
3. Liu C, Xu X, Bai Y, et al. Air pollution-mediated susceptibility to inflammation and insulin resistance: influence of CCR2 pathways in mice. Environ Health Perspect. 2014; 122: 17–26.
4. Maiseyeu A, Badgeley MA, Kampfrath T, et al. In vivo targeting of inflammation-associated myeloid-related protein 8/14 via gadolinium immunonanoparticles. Arterioscler Thromb Vasc Biol. 2012; 32: 962–70.
5. Shah Z, Pineda C, Kampfrath T, et al. Acute DPP-4 inhibition modulates vascular tone through GLP-1 independent pathways. Vascul Pharmacol. 2011; 55: 2–9.
6. Bagalkot V, Badgeley MA, Kampfrath T, Deiuliis JA, Rajagopalan S, Maiseyeu A. Hybrid nanoparticles improve targeting to inflammatory macrophages through phagocytic signals. J Control Release. 2015; 217: 243–55.
7. Chen W, Cormode DP, Vengrenyuk Y, et al. Collagen-specific peptide conjugated HDL nanoparticles as MRI contrast agent to evaluate compositional changes in atherosclerotic plaque regression. JACC Cardiovasc Imaging. 2013; 6: 373–84.
8. Kamaly N, Fredman G, Subramanian M, et al. Development and in vivo efficacy of targeted polymeric inflammation-resolving nanoparticles. Proc Natl Acad Sci U S A. 2013; 110: 6506–11.
9. Vucic E, Sanders HMHF, Arena F, et al. Well-defined, multifunctional nanostructures of a paramagnetic lipid and a lipopeptide for macrophage imaging. J Am Chem Soc. 2009; 131: 406–7.
10. Jain M, Wu B, Pisapia D, Salvatore S, Mukherjee S, Narula N. A component-by-component characterisation of high-risk atherosclerotic plaques by multiphoton microscopic imaging. J Microsc. 2017; 268: 39–44.
11. Henning LN, Azad AK, Parsa KVL, Crowther JE, Tridandapani S, Schlesinger LS. Pulmonary surfactant protein A regulates TLR expression and activity in human macrophages. J Immunol. 2008; 180: 7847–58.
12. Crowther JE, Schlesinger LS. Endocytic pathway for surfactant protein A in human macrophages: binding, clathrin-mediated uptake, and trafficking through the endolysosomal pathway. Am J Physiol Lung Cell Mol Physiol. 2006; 290: L334–42.
13. Vandesompele J, De Preter K, Pattyn F, et al. Accurate normalization of real-time quantitative RT-PCR data by geometric averaging of multiple internal control genes. Genome Biol. 2002; 3: RESEARCH0034.
14. Allain CC, Poon LS, Chan CS, Richmond W, Fu PC. Enzymatic determination of total serum cholesterol. Clin Chem. 1974; 20: 470–5.
15. Fossati P, Prencipe L. Serum triglycerides determined colorimetrically with an enzyme that produces hydrogen peroxide. Clin Chem. 1982; 28: 2077–80.
16. Mog B, Asase C, Chaplin A, Gao H, Rajagopalan S, Maiseyeu A. Nano-Antagonist Alleviates Inflammation and Allows for MRI of Atherosclerosis. Nanotheranostics. 2019; 3: 342–55.
17. Kritharides L, Jessup W, Mander EL, Dean RT. Apolipoprotein A-I-mediated efflux of sterols from oxidized LDL-loaded macrophages. Arterioscler Thromb Vasc Biol. 1995; 15: 276–89.
18. Chaplin A, Gao H, Asase C, et al. Systemically-delivered biodegradable PLGA alters gut microbiota and induces transcriptomic reprogramming in the liver in an obesity mouse model. Sci Rep. 2020; 10: 1–16.
19. Rao X, Zhong J, Maiseyeu A, et al. CD36-dependent 7-ketocholesterol accumulation in macrophages mediates progression of atherosclerosis in response to chronic air pollution exposure. Circ Res. 2014; 115: 770–80.
20. Ying Z, Xie X, Bai Y, et al. Exposure to concentrated ambient particulate matter induces reversible increase of heart weight in spontaneously hypertensive rats. Part Fibre Toxicol. 2015; 12: 15.
21. Ying Z, do Carmo JM, Xiang L, et al. Inhibitor κB kinase 2 is a myosin light chain kinase in vascular smooth muscle. Circ Res. 2013; 113: 562–70.
22. Kuo M-S, Kalbfleisch JM, Rutherford P, et al. Chemical analysis of atherosclerotic plaque cholesterol combined with histology of the same tissue. J Lipid Res. 2008; 49: 1353–63.
23. Maiseyeu A, Mihai G, Roy S, et al. Detection of macrophages via paramagnetic vesicles incorporating oxidatively tailored cholesterol ester: an approach for atherosclerosis imaging. Nanomedicine . 2010; 5: 1341–56.
